# Paraffin Coated with Diatomite as a Phase Change Material (PCM) in Heat Storage Systems—A Review of Research, Properties, and Applications

**DOI:** 10.3390/ma18225166

**Published:** 2025-11-13

**Authors:** Agnieszka Przybek, Maria Hebdowska-Krupa, Michał Łach

**Affiliations:** 1 CUT Doctoral School, Cracow University of Technology, Warszawska 24, 31-155 Cracow, Poland; 2 Faculty of Material Engineering and Physics, Cracow University of Technology, Jana Pawła II 37, 31-864 Cracow, Poland; maria.hebdowska-krupa@pk.edu.pl; 3 Interdisciplinary Center for Circular Economy, Cracow University of Technology, Warszawska 24, 31-155 Cracow, Poland

**Keywords:** paraffin-based phase change materials (PCM), diatomite-supported composites, thermal energy storage (TES), heat and mass transfer, energy-efficient building applications

## Abstract

Paraffin-based phase change materials (PCMs) have emerged as promising candidates for thermal energy storage (TES) applications due to their high latent heat, chemical stability, and low cost. However, their inherently low thermal conductivity and the risk of leakage during melting–solidification cycles significantly limit their practical performance. To address these limitations, numerous studies have investigated composite PCMs in which paraffin is incorporated into porous supporting matrices. Among these, diatomite has garnered particular attention due to its high porosity, large specific surface area, and chemical compatibility with organic materials. Serving as both a carrier and stabilizing shell, diatomite effectively suppresses leakage and enhances thermal conductivity, thereby improving the overall efficiency and reliability of the PCM. This review synthesizes recent research on paraffin–diatomite composites, with a focus on impregnation methods, surface modification techniques, and the influence of synthesis parameters on thermal performance and cyclic stability. The mechanisms of heat and mass transport within the composite structure are examined, alongside comparative analyses of paraffin–diatomite systems and other inorganic or polymeric supports. Particular emphasis is placed on applications in energy-efficient buildings, passive heating and cooling, and hybrid thermal storage systems. The review concludes that paraffin–diatomite composites present a promising avenue for stable, efficient, and sustainable phase change materials (PCMs). However, challenges such as the optimization of pore structure, long-term durability, and large-scale manufacturing must be addressed to facilitate their broader implementation in next-generation energy storage technologies.

## 1. Introduction

Phase change materials (PCMs) are substances characterized by their ability to absorb or release substantial amounts of latent heat during phase transitions, such as from solid to liquid or liquid to gas. These materials are renowned for their capacity to store and release thermal energy, rendering them indispensable in various thermal energy storage applications. This capability is primarily attributed to the distinctive thermal properties of PCMs, including a high heat of fusion and a relatively stable phase change temperature range, which facilitate effective temperature regulation in systems such as buildings, vehicles, and other energy-efficient technologies [1,2].

The mechanisms by which phase change materials (PCMs) facilitate thermal energy storage primarily involve phase transitions between solid and liquid states. During these transitions, PCMs absorb heat during the melting process (transition from solid to liquid) and release heat during solidification (transition from liquid to solid), thereby achieving efficient thermal regulation that minimizes environmental temperature fluctuations [3,4]. Recent advancements have led to the development of shape-stable PCMs (SS-PCMs), which address challenges such as material leakage and corrosion while enhancing thermal performance in applications related to building and insulation materials [5]. These innovations enable the effective integration of PCMs into architectural designs, thereby contributing to improved energy efficiency and thermal comfort [2,6].

The effectiveness and efficiency of phase change materials (PCM) in practical applications are contingent upon their thermal properties, including thermal conductivity, density, and heat capacity. Research has demonstrated that the incorporation of nanoparticle-enriched materials enhances the thermal conductivity of conventional PCM, thereby facilitating more rapid heat transfer and improving the performance of thermal energy systems [1,3]. Various PCM, such as fatty acids, paraffins, and salt hydrates, exhibit distinct phase change properties and thermal behaviors, which influence their appropriateness for specific thermal energy management applications [7].

Phase change materials (PCMs) encapsulated in capsules, particularly in microencapsulated forms, have garnered considerable attention due to their improved thermal efficiency and diminished risk of phase separation and leakage. These materials facilitate enhanced integration with diverse substrates, including textiles and construction materials, thereby broadening the scope of PCM applications in domains necessitating effective thermal regulation [8]. As researchers continue to investigate various formulations and encapsulation methodologies, it is anticipated that the spectrum of available PCMs will expand, enabling tailored solutions for energy storage, thermal comfort, and other heat management requirements. PCMs are pivotal in the domain of thermal energy storage due to their capacity to efficiently regulate temperature through latent heat transformations. Ongoing research and development in PCM properties and formulations hold the promise of future innovations that may further augment their applicability across numerous sectors.

### 1.1. The Importance of Phase Change Materials (PCM) in the Context of Thermal Energy Storage

Phase change materials (PCMs) are integral to thermal energy storage (TES) technologies due to their capacity to absorb and release thermal energy during phase transitions, particularly in solid–liquid transformations. During these transitions, PCMs can store substantial amounts of latent heat at nearly constant temperatures, thereby facilitating efficient thermal regulation across various applications. The integration of PCMs into contemporary energy systems presents numerous benefits, including increased energy density, effective temperature control, and enhanced energy efficiency, rendering them indispensable in both residential and industrial settings.

One of the primary advantages of phase change materials (PCMs) is their superior energy storage density compared to traditional sensible heat storage systems. PCMs are capable of storing a greater amount of heat per unit volume than conventional systems, thereby enabling more compact energy storage solutions without compromising performance [9,10]. Furthermore, research indicates that PCMs can yield substantial energy savings by functioning as thermal buffers, thereby reducing heating and cooling demands in buildings [11,12]. These characteristics not only enhance energy efficiency but also contribute to sustainability by optimizing existing thermal systems.

Furthermore, phase change materials (PCMs) exhibit isothermal characteristics during the phase transition process, maintaining a constant temperature while absorbing or releasing heat. This attribute is particularly advantageous in applications necessitating precise temperature regulation, such as battery thermal management systems, where sustaining a stable thermal environment enhances both performance and durability [13]. Liu et al. elucidate that phase change separators employed in lithium-ion batteries exhibit exceptional heat storage capabilities, thereby improving resistance to thermal shock and enhancing overall safety [13].

Recent advancements have facilitated the development of innovative materials and methodologies that enhance the performance of phase change materials (PCMs). For instance, the advent of nanocapsulated PCMs has improved energy transfer and mitigated issues associated with phase separation, thereby augmenting their efficiency in thermal energy applications [11,14]. Furthermore, the integration of nanomaterials with PCMs can enhance thermal conductivity, thereby expediting energy storage and release processes, which is essential in applications with variable thermal energy supply, such as renewable energy systems [15,16].

The versatility of phase change materials (PCM) encompasses a broad spectrum of applications, ranging from building materials that enhance the energy efficiency of structures [17,18] to cooling systems for high-performance electronics and spacecraft [19,20]. Their capacity to mitigate temperature fluctuations and shift energy demand renders them indispensable in contemporary energy management systems, significantly reducing peak loads on power grids [21]. As global emphasis on renewable energy and environmental sustainability intensifies, the integration of PCM into various sectors represents a promising strategy for managing energy efficiency, while simultaneously addressing issues related to resource consumption and waste management. The significance of PCM in thermal energy storage extends beyond their technical capabilities; they are essential for the advancement of energy management, enhancing system efficiency, and supporting sustainable practices. Ongoing research and development efforts focused on PCM promise a future of even greater efficiency and broader application, underscoring their pivotal role in addressing contemporary energy challenges.

### 1.2. Characteristics of Paraffins as PCM

Paraffin is a widely acknowledged phase change material (PCM) renowned for its effective thermal energy storage capabilities. As a PCM, paraffin wax exhibits several attributes that render it a favorable option for thermal energy management applications, particularly due to its high latent heat of fusion. This property allows paraffin wax to absorb and store substantial amounts of thermal energy during the phase transition from solid to liquid, subsequently releasing this energy during solidification [22,23]. The typical melting point of paraffin wax ranges from 40 °C to 60 °C, rendering it particularly suitable for medium and low-temperature applications [24,25].

The advantages of paraffin as a phase change material (PCM) are manifold. Firstly, it possesses a high latent heat capacity, facilitating the storage of substantial heat within a relatively compact volume. This characteristic is particularly advantageous in applications necessitating long-term temperature regulation, as it enables efficient energy storage and release without significant temperature fluctuations [22,26]. Secondly, paraffin is chemically stable, non-toxic, and cost-effective, rendering it appealing for numerous practical applications [23,25]. Furthermore, it exhibits low vapor pressure, thereby minimizing the risk of evaporation losses during operation, and demonstrates a relatively low degree of supercooling during phase transitions, which is beneficial for maintaining consistent thermal performance [25,27].

Nevertheless, paraffin wax exhibits certain limitations that impact its efficacy as a phase change material (PCM). The primary limitation is its low thermal conductivity, which can impede heat transfer during the charging and discharging cycles. This constraint may result in slower response times in systems that necessitate rapid thermal regulation [24,26]. To enhance the thermal conductivity of paraffin, researchers have explored the incorporation of nanoparticles or other additives, such as expanded graphite or aluminum oxide, which have demonstrated promising potential in significantly improving heat exchange performance [28,29]. Despite these advancements, challenges associated with volume changes during phase transitions—such as leakage and the requirement for specialized containers—can complicate the application of paraffin wax in various contexts [25,30]. Nonetheless, paraffin wax remains a prevalent choice among phase change materials due to its advantageous thermal properties and compatibility with diverse energy storage, cooling, and temperature management systems. Progress in composite materials and encapsulation techniques is anticipated to further enhance the cost-effectiveness and performance of paraffin wax-based phase change materials, thereby creating new opportunities for their utilization in solutions aimed at augmenting energy efficiency.

### 1.3. The Role of Porous Carriers in Stabilizing and Improving the Properties of PCM

Porous carriers are integral to the stabilization and enhancement of phase change materials (PCM), particularly within composite phase change materials (CPCM). The incorporation of porous structures offers numerous functional advantages that improve thermal energy management and enhance operational efficiency. A primary benefit of employing porous carriers is their capacity to prevent the leakage of liquid PCM during phase transitions. As demonstrated by Fang et al., porous materials restrict the flow of the liquid phase, thereby preserving the structural integrity of the composite material even when the PCM transitions to a liquid state [31]. This shape stability enables CPCMs to maintain their macroscopic form, rendering them more practical for various applications, such as lithium-ion battery thermal management systems and other energy storage devices [31].

Moreover, porous supports substantially enhance the thermal conductivity of phase change materials (PCM). The pores within these supports establish efficient thermal pathways that facilitate heat transfer to and from the PCM, thereby reducing the duration required for heat absorption and release during phase change processes. Chen et al. highlight that the pore structure contributes not only to thermal stability but also to the overall enhancement of the thermal performance of form-stable phase change materials (FSPCM) [32]. This characteristic is essential in applications necessitating rapid thermal regulation, such as in building materials and electronic devices [33].

The mechanical strength imparted by the porous supports enhances the reliability of CPCM. This structural reinforcement ensures that the composite can endure mechanical loads without compromising its thermal properties. Liu et al. report that embedding PCM within porous metal or ceramic supports results in phase change materials with robust thermal and mechanical properties, rendering them suitable for medium- and high-temperature applications [34]. Furthermore, the incorporation of fillers and additives into the porous matrix can further augment the overall thermal conductivity of the composite, thereby leading to more efficient thermal energy storage systems [35].

Despite these advantages, the selection of appropriate porous supports is essential for optimizing the efficiency of composite phase change materials (CPCM). Factors such as pore size, shape, and material compatibility with the phase change material (PCM) significantly influence the rate of heat exchange and overall performance. For instance, Zhao et al. emphasize the importance of employing porous materials that remain stable at elevated temperatures, such as titanium dioxide foams, which demonstrate excellent effectiveness in thermal energy storage [36]. Furthermore, optimizing porous structures to enhance heat transfer properties represents a key area for future research, as there is a persistent need to effectively balance stability, durability, and thermal conductivity [31,37]. Porous supports substantially improve the stabilization and properties of phase change materials, rendering them more suitable for various applications in thermal energy storage and management. Their capacity to prevent leakage, enhance thermal conductivity, and provide mechanical strength underpins the development of advanced composite materials tailored to contemporary energy requirements.

### 1.4. Why Diatomite as a Matrix Material for PCM Production?

Diatomite is increasingly utilized as a matrix material for the production of phase change materials (PCM) due to its exceptional properties, including availability, high porosity, low cost, and chemical compatibility. These attributes enhance the effectiveness of diatomite in applications related to thermal energy storage. Diatomite, also referred to as diatomaceous earth, is a naturally occurring, amorphous siliceous material formed from the fossilized remains of diatoms. It is found in significant deposits across various geographic regions, including substantial reserves in the United States, China, and Algeria [38,39]. This widespread availability renders diatomite a practical choice as a matrix material for PCM, ensuring a reliable supply chain for industrial applications.

One of the defining characteristics of diatomite is its remarkably high porosity, often surpassing 80–90% [38,40]. This porosity not only facilitates a substantial absorption capacity but also offers adequate structural space to encapsulate phase change materials (PCM). The porous architecture enhances thermal conductivity and the rate of heat exchange, both of which are essential for the effective performance of PCM [38,41]. For instance, high porosity assists in the encapsulation of phase change materials, enabling larger quantities of material to be accommodated within a smaller volume, which is vital in applications necessitating efficient temperature regulation [42,43].

Diatomite is generally more cost-effective compared to other advanced materials utilized in PCM applications. Its affordability can substantially lower the overall expenses associated with temperature management systems, rendering it an appealing choice for manufacturers [38,39]. The economical nature of diatomite does not compromise its quality; instead, it offers a viable solution for enhancing the heat storage capacity of composite materials while maintaining economic feasibility [44,45].

Diatomite demonstrates remarkable chemical inertness, which enhances its compatibility with various polymers and organic phase change materials (PCMs). This property permits the efficient integration or impregnation of PCMs into diatomite matrices without undesirable reactions, thereby augmenting the stability and performance of the resultant composites [38,44]. The compatibility of diatomite with other materials facilitates a broad spectrum of applications, ranging from thermal energy storage systems in buildings to cooling applications in electronic devices, without the risk of degradation [46,47]. Furthermore, the exceptionally large surface area of diatomite contributes to the formation of stable interfaces within the composite, potentially leading to enhanced mechanical and thermal properties [39,48].

Diatomite presents itself as a highly promising matrix material for the production of phase change materials (PCM) due to its abundant availability, high porosity that facilitates efficient thermal management, cost-effectiveness that supports economic viability, and compatibility that enhances the performance of composite solutions for thermal energy storage. Its application can significantly contribute to advancements in thermal energy storage technologies, potentially leading to a wider adoption of PCM across various industries.

While numerous reviews have investigated the application of phase-change materials (PCMs) for thermal energy storage, the majority have focused on general PCM classification or enhancing thermal conductivity through nanofillers. Nonetheless, a distinct research gap persists regarding paraffin–diatomite composite systems, which integrate organic PCMs with porous mineral carriers. Previous studies have seldom offered a comprehensive comparison of preparation techniques or explored the interdependence between material structure, processing methods, and performance outcomes. Furthermore, the existing literature inadequately addresses the contradictions between thermal conductivity enhancement, latent-heat retention, and long-term stability, which currently impede the large-scale application of such materials. Consequently, this review seeks to address this gap by systematically evaluating fabrication approaches, correlating structural parameters with key thermal properties, and identifying the primary technical barriers that must be overcome for industrial implementation. The novelty of this work lies in establishing a coherent correlation framework linking material design strategies—such as surface modification, hybridization, and pore-structure control—with performance indicators including thermal conductivity, latent-heat capacity, cyclic durability, and cost-effectiveness. The paper is structured to provide a logical progression from the fundamental characteristics and preparation methods of paraffin–diatomite PCMs to their performance evaluation, comparative analysis, and discussion of major challenges and future research directions.

## 2. Properties of Paraffin as PCM

### 2.1. Thermal Parameters

Paraffin wax is extensively utilized as a phase change material (PCM) due to its advantageous thermal properties, which enable effective thermal energy storage. The primary thermal parameters of paraffin wax as a PCM include its melting temperature, heat capacity, and phase change enthalpy, all of which are critical for its implementation in temperature management systems.

The melting point of paraffin is contingent upon its specific composition, typically ranging from approximately 30 °C to 90 °C. This range renders paraffin an optimal candidate for various applications, particularly in heating and cooling systems designed to maintain a comfortable indoor temperature [49,50]. For instance, certain paraffins possess a melting point of around 28.2 °C, making them particularly suitable for use in passive solar heating systems and building materials [51]. The capacity to modify the melting point by selecting the length of the paraffin chain or mixtures enables the customization of solutions for diverse temperature management tasks.

The specific heat capacity of paraffin is approximately 2100 J/kg·K when the material is in its solid phase [52]. This parameter is critically important as it quantifies the amount of heat required to elevate the temperature of paraffin, thereby determining its efficacy as a thermal energy carrier. The heat capacity may exhibit slight variations depending on the precise composition of the paraffin and the additives incorporated, which can also influence the overall performance and functionality of the PCM system.

The latent heat of fusion of paraffin is notably high, typically ranging from 180 to 230 kJ/kg [53,54]. This substantial capacity for latent heat storage enables paraffin to effectively absorb and store energy during the melting process and subsequently release it during solidification. These characteristics render paraffin wax a highly desirable phase change material (PCM) for thermal energy storage applications, as it can significantly contribute to energy savings in heating and cooling systems [55]. Furthermore, paraffin wax exhibits minimal to no supercooling during solidification, thereby enhancing its reliability and efficiency as a thermal energy storage material [50].

Paraffin wax exhibits significant thermal properties, including an advantageous melting point, substantial heat capacity, and high phase change enthalpy, which collectively render it an effective phase change material for thermal energy storage systems and temperature regulation. Ongoing advancements in formulations and composite materials continue to enhance the performance characteristics of paraffin-based PCMs, thereby broadening their potential applications. Table 1 provides a summary of the physicochemical properties of selected, commonly used paraffins, including their melting temperature, heat of fusion, and thermal conductivity.

### 2.2. Chemical and Thermal Stability

Paraffin wax serves as an effective phase change material (PCM) primarily due to its remarkable chemical and thermal stability, which are essential for sustaining performance in thermal energy storage applications. A comprehensive understanding of these properties is vital for evaluating the suitability of paraffin in diverse applications, including energy management in buildings, thermal insulation, and renewable energy systems.

Paraffin wax demonstrates remarkable chemical stability, characterized by its non-reactivity and inertness towards most other substances. This stability ensures that paraffin retains its structural integrity and functional performance over extended periods, thereby resisting degradation that could otherwise result in phase separation or reduced efficiency in energy storage applications [60,61]. Furthermore, paraffin can be incorporated into various compositions without posing a risk to packaging materials or adversely affecting adjacent components. This chemical inertness is particularly essential in applications involving diverse building materials and alloys, where material compatibility is of paramount importance.

Thermal stability is a critical parameter influencing the performance of phase change materials (PCM) during heating and cooling cycles. Paraffin exhibits remarkable thermal stability, rendering it resistant to phase change cycles without substantial degradation over time. Extensive research has demonstrated that paraffin can endure numerous melting and freezing cycles without a loss in latent heat capacity [60]. The stability of temperature cycles ensures that paraffin retains its energy storage properties and does not decompose under typical operating conditions. For instance, encapsulated paraffin composites preserve their thermal performance and structural integrity even at elevated temperatures [62].

During the phase transition, paraffin demonstrates minimal to no supercooling, thereby enhancing its reliability as a thermal energy carrier [63]. In paraffin wax, the phenomenon of self-crystallization is observed, facilitating uniform solidification without the formation of disruptive crystalline defects that could compromise its performance [63]. This characteristic is essential for ensuring predictable performance and efficient energy release, aligning with the desired operational properties of the phase change material (PCM).

Despite its low volatility, paraffin wax can experience phase transitions accompanied by volume changes of approximately 10% during melting and cooling processes [64]. If not adequately contained, this may result in leakage. Nevertheless, advancements in encapsulation techniques and the development of shape-stable paraffins have effectively mitigated these challenges, resulting in stable composites that resist leakage while maintaining superior thermal performance [65,66].

The chemical and thermal stability of paraffin wax render it an exceptionally advantageous phase change material for a variety of applications in thermal energy storage. Its capacity to endure thermal cycles while preserving its latent heat properties, coupled with its non-corrosive nature and self-nucleating characteristics, reinforces the suitability of paraffin as a dependable material for enhancing energy efficiency across diverse environments.

### 2.3. Problems: Leakage, Low Thermal Conductivity, Degradation over Multiple Cycles

**Figure 1:**
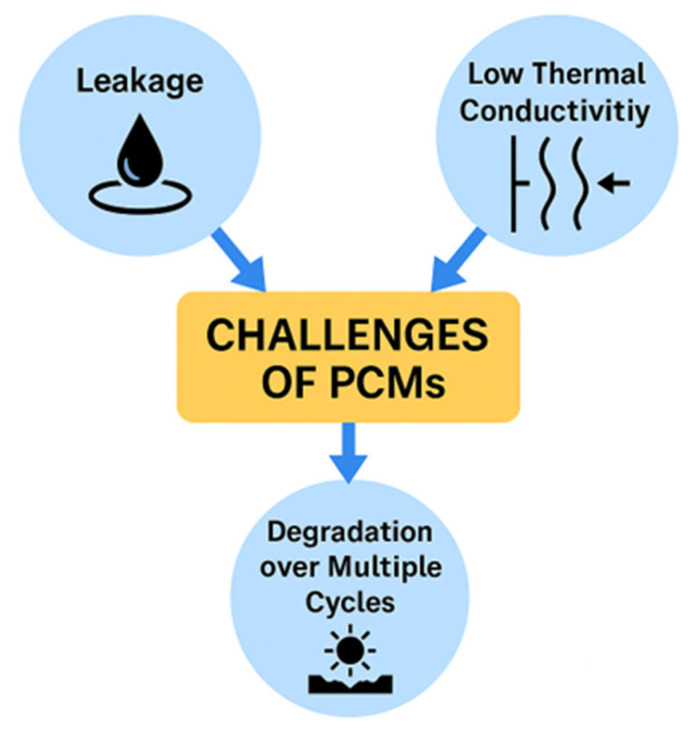
A schematic representation of the key challenges related to phase change materials (PCM), including leakage during phase transition, low thermal conductivity, and degradation after multiple thermal cycles.

Paraffin is a widely utilized phase change material (PCM) that possesses several advantageous properties for thermal energy storage applications. Nevertheless, it is also associated with certain challenges, particularly concerning leakage, low thermal conductivity, and potential degradation after numerous thermal cycles. A comprehensive understanding of these issues is essential for optimizing the performance and application of paraffin as a PCM. Figure 1 presents a diagram illustrating common problems encountered when employing paraffin as a PCM.

One of the primary challenges associated with the utilization of paraffin as a phase change material (PCM) is its propensity to leak during phase transitions. Paraffin experiences substantial volumetric changes, approximately 10% during melting, and can flow in its liquid state, which poses difficulties in retaining it within PCM systems [10,12]. For instance, research has demonstrated that composite materials, such as paraffin combined with porous carriers like expanded graphite, effectively mitigate leakage by enhancing the structural stability of the PCM, thereby preventing liquid escape during thermal cycles and preserving thermal performance [10,12]. In paraffin-based systems, innovative encapsulation techniques are also employed to prevent leakage, thereby enhancing their overall functionality and reliability [67].

Another notable limitation of paraffin is its low thermal conductivity, which, according to general data, ranges from 0.21 to 0.24 W/m·K [68,69]. This low thermal conductivity results in inefficient heat transfer, thereby constraining the rate at which thermal energy can be absorbed or released during phase change processes. Consequently, this presents a challenge for dynamic applications that necessitate rapid thermal response [70]. To mitigate this issue, various strategies have been employed, including the incorporation of materials with high thermal conductivity—such as metallic foams, graphite, or nanomaterials—into the paraffin matrix. These enhancements have been demonstrated to significantly improve the overall thermal performance of paraffin as a PCM [68,71]. By selectively increasing the thermal conductivity of paraffin composites, researchers aim to optimize heat exchange processes without compromising the latent heat storage properties.

Repeated thermal cycling can result in the degradation of paraffin properties, as documented in various studies [12,72]. While paraffin generally remains stable during the melting and solidification phases, extended use under conditions of repeated thermal loading may lead to a reduction in its latent heat storage capacity and potential issues with phase separation. Research suggests that microencapsulation and the incorporation of shape-stable composites can effectively mitigate degradation, enabling paraffin to maintain its thermal energy storage capacity over numerous cycles [72,73]. Furthermore, the addition of metal nanoparticles or carbon-based materials can enhance thermal stability, thereby increasing the resistance of paraffin-based PCMs to thermal degradation and prolonging their lifespan [73,74].

Paraffin wax, while offering notable advantages as a phase change material—such as high latent heat of fusion and widespread availability—encounters challenges including leakage, low thermal conductivity, and degradation over time. Current research into paraffin composites and encapsulation techniques seeks to address these limitations, thereby enhancing the utility and efficacy of paraffin in thermal energy storage systems.

## 3. Characteristics of Diatomite as a PCM Carrier

### 3.1. Structure and Morphology

Diatomite, commonly referred to as diatomaceous earth, is increasingly acknowledged as a promising carrier material for phase change materials (PCM) due to its distinctive structure and morphology, which enhance its efficacy in thermal energy storage applications. Its primary attributes include a microporous and mesoporous structure, along with a substantial specific surface area. Diatomite is characterized by a well-developed porous structure, comprising both micropores (<2 nm) and mesopores (2–50 nm) [43,48]. This hierarchical pore structure significantly influences the effective surface area and adsorption capacity of diatomite, enabling it to effectively accommodate PCM and facilitate heat transfer. The microporosity provides sites for the adsorption of small molecules, while the mesoporosity enhances the material’s capacity for fluid transport, thereby supporting the movement of PCM during phase change processes [43]. This dual porosity is essential for maintaining the thermal performance and stability of composite phase change materials, thereby enhancing the efficiency of thermal energy storage systems [75].

The specific surface area of diatomite can be substantial, frequently surpassing 200 m^2^/g, contingent upon the type and processing methods employed [76]. An increased specific surface area enhances the interaction between the phase change material (PCM) and the diatomite matrix, resulting in improved material infiltration and reduced PCM leakage during phase transitions. This characteristic is essential in the production of SS-PCMs, where the PCM is encapsulated within the porous structure of diatomite, thereby preventing leakage while maintaining its thermal properties across multiple thermal cycles [43,48].

The distinctive structural characteristics of diatomite significantly contribute to its mechanical strength and thermal stability. Its porous nature facilitates weight reduction while preserving mechanical integrity, thereby allowing its application in construction materials without compromising structural properties [77]. Nevertheless, it is important to acknowledge that although diatomite enhances PCM adsorption, its inherently low thermal conductivity may restrict the overall thermal performance of the composite during periods of rapid heating or cooling. Current research endeavors aim to mitigate this limitation by incorporating materials or additives that can improve thermal conductivity within the diatomite matrix [48,78].

**Figure 2:**
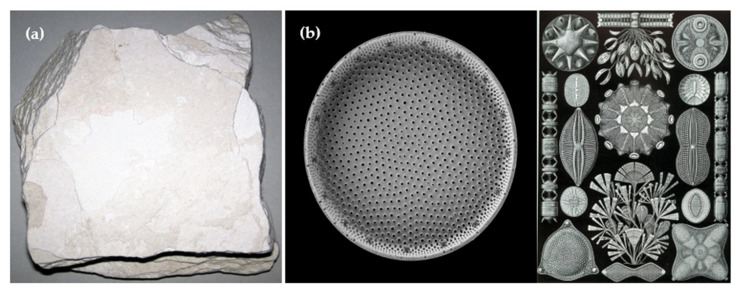
(**a**) diatomite in rock form, presenting a natural aggregation of diatom remains [79], (**b**) individual diatoms (silica shells) seen under a microscope, showing their characteristic structure and morphology [80,81].

The structural and morphological characteristics of diatomite, which include microporous and mesoporous networks as well as a substantial specific surface area, render it an effective carrier material for phase change materials (PCMs). These attributes enhance diatomite’s capacity to absorb, retain, and release thermal energy, thereby augmenting the overall thermal properties of diatomite-based PCMs. As research continues to focus on enhancing the thermal conductivity and stability of diatomite composites, their application in energy storage and management systems is anticipated to expand, offering efficient and sustainable solutions. Figure 2 illustrates a comparison of two forms of diatomite material. On the left, diatomite is depicted in its rock form, which is a sedimentary deposit primarily composed of calcareous and siliceous remains of diatoms. On the right, individual diatoms are shown under a microscope, revealing their characteristic silica shell and diverse morphology. This comparison provides a more comprehensive understanding of both the macroscopic form of diatomite as a rock and the microscopic structure of its fundamental components—the diatoms.

### 3.2. Physico-Chemical Properties

Diatomite, commonly referred to as diatomaceous earth, exhibits several significant physical and chemical properties that enhance its utility as a carrier material in phase change material (PCM) applications. Notable characteristics include its hydrophilicity, mineral composition—particularly its high silica content—and distinctive structural properties, all of which contribute to its efficacy as a PCM carrier.

Diatomite demonstrates notable hydrophilic characteristics attributable to its extensive specific surface area and porous architecture. The surface of diatomite predominantly consists of siliceous frustules, which incorporate polar functional groups that enhance its affinity for water [82]. This hydrophilicity augments diatomite’s capacity to adsorb and retain moisture, rendering it advantageous in applications necessitating humidity regulation in conjunction with temperature management. Its water retention capability enables diatomite to be employed in various contexts, such as an additive in building materials, where it can enhance overall humidity control and energy efficiency.

The primary mineral constituent of diatomite is biogenic silica (SiO_2_), which typically comprises over 60% of its composition [44,83]. This substantial silica content is essential for its favorable thermal and structural properties. Diatomite frequently contains significant quantities of amorphous silica, contributing to its lightness, low bulk density, and high specific surface area [84]. The presence of silica not only ensures structural integrity but also enhances its stability in various chemical environments, rendering it an excellent candidate for use in temperature management systems where stability under diverse conditions is required.

Diatomite is distinguished by its highly developed porous structure, characterized by a substantial specific surface area, often surpassing 200 m^2^/g [85]. This pronounced internal porosity is essential as it facilitates the adsorption of phase change materials (PCMs), thereby enabling efficient storage and release of thermal energy during phase change processes. The hierarchical structure comprises both micro- and mesopores, which enhance interactions with the adsorbed PCMs and augment thermal performance by permitting rapid heat transfer within the material [47]. Furthermore, the extensive pore network aids in minimizing the leakage of the PCM liquid phase, thus enhancing the efficiency and reliability of composite thermal energy storage systems [86].

The hydrophilic properties of diatomite, in conjunction with its high silica content and extensive porous structure, make it an ideal carrier for phase change materials (PCMs). These characteristics enhance the material’s heat adsorption capacity, optimize thermal management, and maintain structural integrity under diverse thermal conditions. Consequently, the integration of these features positions diatomite as an effective supporting material that enhances the performance of PCMs in various applications, including building materials, renewable energy systems, and thermal energy storage devices.

### 3.3. The Ability to Absorb Paraffin and Its Role in Reducing Leaks

Diatomite is an effective carrier material for phase change materials (PCMs) due to its distinctive properties, which enhance paraffin absorption and minimize leakage during thermal cycles. The key properties of diatomite in this context are examined below, with a focus on its efficacy in capturing paraffin and the associated benefits. Diatomite demonstrates an exceptionally high paraffin absorption capacity, which is essential for its application as a PCM carrier. Its porous structure, characterized by numerous micropores and mesopores, facilitates significant infiltration of paraffin into its matrix [40,45]. This process typically involves the direct impregnation method, wherein diatomite is combined with molten paraffin, allowing the PCM to effectively occupy the pores and cavities within the diatomite matrix. Research indicates that modifying diatomite through calcination can enhance its absorption capacity, enabling the capture of larger quantities of paraffin [45]. This high level of absorption ensures the storage of sufficient PCM, thereby facilitating effective temperature management, as PCM can release or absorb thermal energy in a more controlled manner.

The porous architecture of diatomite plays an essential role in effectively mitigating leaks, a prevalent issue in conventional paraffin systems. During the phase transitions of paraffin wax, specifically melting and solidification, substantial volumetric changes can occur, potentially resulting in leaks if not adequately contained [43]. However, the incorporation of diatomite as a carrier facilitates the retention of paraffin within its porous structure, thereby significantly reducing leaks during operation.

The encapsulation of paraffin within the diatomite structure not only stabilizes the phase change material (PCM) but also prevents the leakage of liquid paraffin from the matrix, thereby ensuring the PCM’s effectiveness over multiple thermal cycles [40,87]. This characteristic is particularly advantageous in applications where consistent performance during heating and cooling cycles is essential. By minimizing leakage, diatomite enhances the reliability and efficiency of PCM systems, ultimately improving energy storage performance and rendering this technology more practical for diverse applications. Diatomite functions as a highly effective support material for paraffin wax, with its extensive porous structure facilitating a high absorption capacity. Furthermore, its ability to minimize leakage during phase change processes enhances the overall reliability and efficiency of thermal energy storage systems. Ongoing innovations in diatomite modification and its integration with various PCMs continue to expand its application in energy management solutions.

### 3.4. Comparison with Other Carrier Materials

Diatomite has emerged as a significant carrier material for phase change materials (PCM) due to its distinctive physical and chemical properties. In comparison to other widely used carrier materials such as zeolites, perlite, silica, clays, and bentonite, diatomite offers unique advantages that can enhance the performance of PCM in thermal energy storage applications.

Diatomite demonstrates a substantial capacity for paraffin absorption, attributable to its porous structure, which comprises a network of micropores and mesopores. This structural characteristic facilitates the effective penetration of phase change materials (PCM), enabling diatomite to retain considerable quantities of paraffin. Empirical studies have indicated that diatomite can efficiently absorb paraffin, thereby forming form-stable phase change materials with enhanced temperature management properties [88]. In comparison, other materials, such as perlite, also function as carriers for PCM; however, the absorption capacity of expanded perlite for paraffin is 80.1 kJ/kg, which is inferior to that of diatomite [88].

The hydrophilic nature of diatomite enhances its capacity to interact with various phase change materials (PCMs). The substantial silica content, predominantly in amorphous form, significantly contributes to the chemical stability of diatomite and offers a chemically inert surface that effectively engages with PCMs. Modifications to diatomite can further augment its compatibility with specific PCMs and ensure its adaptability to diverse chemical compositions [88]. In contrast, zeolites are distinguished by their high chemical stability and extensive specific surface area, attributed to their crystalline structure. However, their efficacy in adsorbing organic PCMs, such as paraffin, is generally inferior to that of diatomite [89].

Diatomite possesses a high specific surface area, typically exceeding 200 m^2^/g, which enhances thermal interactions and facilitates effective heat transfer during the phase change processes of PCMs. Research indicates that PCMs incorporating diatomite demonstrate advantageous thermal properties due to this high specific surface area, which contributes to improved thermal conductivity and energy storage efficiency [88]. While other materials, such as silica and bentonite, also offer a reasonable specific surface area, their structures may not support the retention of liquid PCMs as effectively as diatomite, particularly under conditions of high thermal cycling [90].

The porous structure of diatomite is instrumental in mitigating leakage during phase transitions, a prevalent issue for many organic phase change materials (PCMs) during melting. The absorptive capacity and structural characteristics of diatomite facilitate the retention of paraffin, thereby minimizing leakage risk and stabilizing the composite material amid temperature fluctuations [88]. Conversely, other carriers, such as expanded graphite and silica, may experience leakage challenges during extreme thermal cycles, although they can also enhance thermal conductivity to some extent [86].

Diatomite is distinguished as an effective carrier material for phase change materials (PCM), particularly when compared to alternatives such as zeolites, perlite, silica, clays, and bentonite. Its superior paraffin absorption capacity, extensive specific surface area, hydrophilicity, and capacity to minimize leakage render it especially suitable for phase change applications. Ongoing research aims to further investigate and harness the unique properties of diatomite, potentially leading to enhanced formulations and applications in thermal energy management solutions. Table 2 provides a comparative analysis of the structural characteristics, typical PCM content, thermal conductivity, leakage resistance, chemical compatibility, and stability, as well as typical applications of diatomite and other PCM carriers such as zeolites, perlite, silica, or clays.

## 4. Methods of Producing Paraffin PCM Composites with Diatomite

### 4.1. Physical Methods: Vacuum Impregnation, Capillary Adsorption

The utilization of physical methods for the production of paraffin composite phase change materials (PCM) with diatomite markedly enhances thermal energy storage capabilities while preserving structural integrity. Two prominent techniques in this domain are vacuum impregnation and capillary adsorption, which enable the effective integration of paraffin into diatomite, thereby optimizing its thermal properties [65,97,98].

Vacuum impregnation is a well-established technique for the synthesis of diatomite–paraffin composites. This process entails placing diatomite within a vacuum chamber, where paraffin is drawn into the porous structure of the diatomite under conditions of reduced pressure. Empirical studies have demonstrated that this method significantly enhances the incorporation of phase change materials (PCM) into the diatomite matrix, thereby yielding composites with improved phase change properties [99,100]. For instance, Jeong et al. successfully prepared composites wherein vacuum impregnation facilitated efficient PCM loading and enhanced shape stabilization of the PCM within the diatomite, thereby contributing to a more reliable heat storage capacity [99]. Furthermore, this method frequently results in a reduced supercooling temperature during solidification, which is a critical factor for effective temperature management [101].

Capillary adsorption represents an effective physical technique for the synthesis of paraffin–diatomite composites. This method leverages the capillary forces inherent in the porous structure of diatomite, facilitating the absorption of paraffin into the pores without necessitating vacuum application. Research has demonstrated that employing capillary action for adsorption can markedly enhance the efficiency of paraffin loading in diatomite, thereby offering a substantial specific surface area for heat exchange [38,42]. Empirical evidence suggests that the porous architecture of diatomite not only effectively retains paraffin but also contributes to the retention of latent heat, rendering the resulting composites more efficacious for heat management applications [42]. The interactions between diatomite and paraffin, augmented by capillary forces, ensure the retention of the phase change material (PCM) in situ, thereby enhancing the overall stability and performance of the energy storage system [41].

Moreover, the integration of vacuum impregnation and capillary forces can further enhance the preparatory processes. By capitalizing on the benefits of both techniques, researchers have achieved notable improvements in the thermal conductivity and latent heat capacity of the resulting composites. For instance, Konuklu et al. demonstrated that the combination of these methods can yield high-performance composites with superior thermal properties, resulting in a substantial increase in the product’s energy storage capacity [43,101].

The application of vacuum impregnation and capillary adsorption in the synthesis of diatomite–paraffin composites is instrumental in augmenting their efficacy as viable solutions for thermal energy storage. The meticulous selection and implementation of these physical techniques yield composites with enhanced thermal stability, diminished supercooling, and elevated heat capacity, rendering them suitable for diverse energy management applications.

### 4.2. Chemical Modifications

The enhancement of composite materials incorporating paraffin as a phase change material (PCM) through the addition of diatomite can be substantially achieved via various chemical modification techniques. A particularly effective approach is silanization, which entails coating the diatomite surface with silane-based coupling agents. This process improves compatibility with paraffin and augments the overall thermal performance of the composites.

The silanization of diatomite enhances the compatibility of the filler with the paraffin matrix. Research indicates that surface modification using silane agents results in improved interfacial adhesion between diatomite and paraffin, which is essential for achieving uniform dispersion of the PCM within the composite material [102]. This approach also contributes to a reduction in water absorption, thereby leading to more stable composite structures over time, as effective interaction at the filler-matrix interface mitigates moisture penetration [44].

Research has demonstrated that calcined diatomite serves as an exceptional sorbent for paraffins, achieving significant PCM loading ratios. Specifically, diatomite subjected to elevated temperatures ranging from 450 °C to 650 °C exhibits remarkable sorption properties attributable to its porous structure, which facilitates the effective encapsulation of paraffin [43]. Post-silanization, diatomite can further augment its sorption capacity and thermal stability, thereby offering dual advantages for composite performance in thermal energy storage applications.

Numerous scholars have highlighted the advantages of employing modified diatomite composites in phase change material (PCM) applications. For instance, following thermal analyses and stability assessments, composites incorporating silanized diatomite exhibited superior thermal response, enhanced latent heat storage, and diminished supercooling effects in comparison to their unmodified counterparts [88,103]. The incorporation of diatomite not only aids in thermal regulation but also serves as a nucleating agent, thereby further facilitating the phase change processes encapsulated in paraffin [104].

The silane modification technique has been effectively employed in numerous studies to enhance the mechanical and thermal properties of composite materials. Surface modification has led to a notable increase in thermal stability and an improvement in structural integrity, which are essential for applications necessitating long-term durability [102,105,106]. By optimizing the silanization process, researchers have demonstrated that such modifications can result in a significant enhancement in the performance of paraffin–diatomite composites under various thermal conditions.

The silanization of diatomite surfaces not only enhances compatibility with paraffins but also improves thermal efficiency, sorption properties, and the stability of the composite. Consequently, this technique is essential for the development of high-performance paraffin phase change material (PCM) composites that are suitable for efficient thermal energy storage.

### 4.3. Hybrid Approaches: Combining Diatomite with Other Materials (Graphene, Activated Carbon, Metals, Polymers)

The hybrid methodology for synthesizing paraffin phase change material (PCM) composites with diatomite has demonstrated a notable enhancement in thermal energy storage efficiency. By integrating diatomite with various substances, including graphene, activated carbon, metals, and polymers, researchers have engineered composites that exhibit improved thermal stability, mechanical properties, and latent heat retention.

Graphene has emerged as a compelling addition to phase change material (PCM) composites, attributed to its remarkable thermal conductivity and mechanical strength. Numerous studies have demonstrated that the integration of graphene into diatomite–paraffin composites enhances their thermal properties. For instance, Li et al. examined paraffin-graphene composites, revealing that this hybrid system offers superior heat storage capacity and an increased latent heat of phase transition [107]. Additionally, Ye et al. developed core–shell structures comprising graphene aerogel encapsulating paraffin, resulting in composites with stable shape and notable heat storage characteristics [108]. These graphene inclusions improve thermal conductivity and may potentially mitigate issues associated with supercooling in PCM applications [109].

Carbon nanotubes have similarly been utilized to develop hybrid diatomite composites. Han et al. demonstrated that the paraffin-A-CNT composite exhibited remarkable cyclic stability during phase transitions over more than 300 cycles, indicating its durability in practical applications [65]. The incorporation of carbon nanotubes enhances heat storage capacity by improving interfacial interactions between the paraffin and the filler material, thereby augmenting the total latent heat of the composite [110].

Activated carbon, recognized for its extensive surface area and porous architecture, has been integrated into diatomite composites. This combination results in enhanced thermal conductivity and structural integrity. The capacity of activated carbon to absorb heat effectively complements the stability afforded by diatomite, which is essential in applications necessitating consistent temperature regulation. Research indicates that the incorporation of activated carbon significantly augments the overall efficacy of thermal energy storage systems [65].

Research has demonstrated that the integration of diatomite with polymer matrices enhances mechanical properties and mitigates the leakage of molten phase change materials (PCM). For instance, Sun and Xiao elucidated the synthesis of microcapsules featuring a hybrid polymer shell, illustrating how these structures can stabilize phase change materials and sustain their efficacy across numerous thermal cycles [104]. This polymer reinforcement, in conjunction with diatomite, facilitates the attainment of a stable form while optimizing thermal energy retention capabilities.

Furthermore, metals play a significant role in enhancing the thermal properties of composites. The integration of metallic fillers into the diatomite matrix can significantly improve heat conduction characteristics. This incorporation facilitates efficient heat transfer, rendering the resulting composites highly suitable for applications in high-performance thermal energy storage [111].

**Figure 3:**
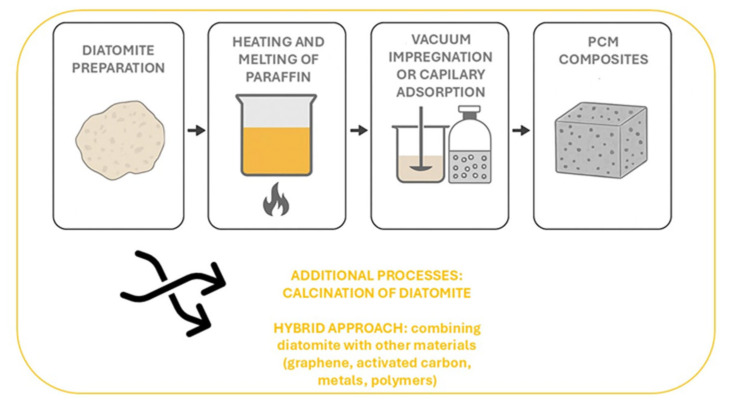
Diagram of the diatomite impregnation process with paraffin—presents the subsequent stages of preparing the PCM composite: preparation of the diatomite raw material, heating and melting of paraffin, capillary adsorption or vacuum impregnation process, followed by obtaining a composite material with paraffin evenly distributed in the pores of the diatomite structure.

Hybrid methodologies that integrate diatomite with graphene, carbon nanotubes, activated carbon, polymers, and metals constitute a comprehensive strategy for enhancing the performance of paraffin-based phase change material (PCM) composites. These approaches result in improved thermal stability, increased latent heat storage capacity, and facilitate innovative advancements in thermal energy management technologies. Figure 3 provides a summary of the methods employed for impregnating diatomite with paraffin.

### 4.4. Comparative Analysis of Preparation Methods

The three preparation techniques discussed—physical methods (vacuum impregnation and capillary adsorption), chemical modification (primarily silanization and calcination), and hybrid approaches (integrating diatomite with graphene, activated carbon, polymers, or metals)—exhibit substantial differences in process conditions, achievable loading efficiency, and resultant thermal parameters. Table 3 provides a summary of the principal parameters and performance indicators documented in the literature for these methods, including enhancements in thermal conductivity and latent heat retention.

In summary, chemical modification provides an optimal balance between high paraffin loading and structural stability. Concurrently, hybrid composites achieve the most significant enhancement in thermal conductivity, with improvements of up to 60–70% compared to unmodified paraffin–diatomite systems. Physical impregnation remains the most straightforward and cost-effective technique, suitable for large-scale applications where moderate thermal performance is sufficient. A comparative evaluation of these methods clearly indicates that the optimal approach is contingent upon the intended application; building materials benefit from the cost-effectiveness and shape stability of physically impregnated composites, whereas high-performance thermal storage systems derive the greatest advantage from chemically modified or hybrid materials with enhanced thermal conductivity and cyclic durability.

## 5. Thermal and Functional Properties of Paraffin–Diatomite Composites

### 5.1. Temperature Stability and Energy Retention

Paraffin–diatomite composites have emerged as promising phase change materials (PCMs) for thermal energy storage, particularly in applications related to energy conservation in buildings and temperature regulation [117,118,119,120,121]. The distinctive porous structure of diatomite, especially following purification and calcination, significantly enhances its capacity to adsorb paraffin, resulting in composites with a high paraffin encapsulation ratio and stable phase change behavior [117,118,119,122,123]. For instance, the fusion adsorption of paraffin with calcined diatomite yields composites with a phase change temperature of approximately 33 °C and latent heat values nearing 89.5 kJ/kg, demonstrating both thermal reliability and chemical stability over repeated cycles [117,118,124,125,126].

Surface modification of diatomite, such as through oleophobic treatment, significantly enhances encapsulation efficiency, achieving a rate of 84.5%, and reduces leakage during the phase transition of paraffin from solid to liquid states [118,119,127,128]. These modifications also contribute to the increased thermal reliability of the composite, as demonstrated by minimal degradation after 50 or more melting/freezing cycles [118,119,129]. Simulation studies indicate that the integration of such composites into building components, such as windows, can lower indoor temperatures by over 5 °C compared to traditional materials, underscoring their potential in energy-saving applications [118,119,130,131].

The application of paraffin–diatomite composites extends to coatings and mortars. Notably, modified diatomite–paraffin coatings exhibit remarkable thermal stability and minimal leakage, even after undergoing 500 thermal cycles [118,132]. When these coatings are applied to building walls, they effectively maintain the phase change temperature within the range of 26–28 °C, reduce temperature fluctuations to a mere 2.5 °C, and decrease cooling loads by approximately 19% during the summer months, thereby achieving substantial energy savings [118,120,121,133].

Extensive reviews and experimental investigations have demonstrated that the incorporation of property-enhancing materials, such as expanded vermiculite or nanomaterials, can significantly augment the thermal conductivity, mechanical strength, and shape stability of paraffin-based composites [120,121,122,123,134]. These enhancements do not adversely affect the latent heat storage capacity, which remains substantial, and the composites preserve their structural and chemical integrity following rigorous thermal cycling [120,121,122,135]. In lightweight mortars, the application of paraffin-vermiculite-diatomite composites can mitigate indoor temperature fluctuations by 3.6 °C, thereby providing effective thermal regulation throughout the year [121,133,136].

Numerical and experimental analyses indicate that the integration of these composites into building materials can reduce peak wall temperatures by up to 2.7 °C and achieve energy savings exceeding 17% in comparison to conventional cement mortars [120,121,133]. The combination of high latent heat, adjustable phase change temperatures, and robust cyclic stability renders paraffin–diatomite composites particularly suitable for practical applications in thermal energy storage and temperature regulation within buildings [117,118,119,120,121,122,123,124].

Recent advancements in the design and modification of paraffin–diatomite composites have resulted in the creation of materials exhibiting exceptional thermal stability, high energy retention, and robust functionality in practical applications. These developments are substantiated by both laboratory and simulation studies, which confirm the potential of these composites for extensive use in energy-efficient building systems [117,118,119,120,121,122,123,124].

### 5.2. Thermal Conductivity (And Ways to Improve It)

The thermal conductivity of paraffin–diatomite composites is a critical determinant of their efficacy in thermal energy storage applications. Diatomite, noted for its unique porous structure, generally exhibits low thermal conductivity, which can pose a limitation when incorporated into composites. Nonetheless, various strategies have been identified to enhance the thermal conductivity of these composites, thereby augmenting their effectiveness as phase change materials (PCM).

Research has demonstrated that the incorporation of nanofillers, such as carbon nanotubes (CNT) and graphene, markedly enhances the thermal conductivity of paraffin–diatomite composites. Specifically, it has been documented that the thermal conductivity of paraffin and diatomite mixtures can be augmented by 42.45% with the addition of merely 0.26 wt.% of carbon nanotubes [137]. Similarly, the introduction of graphene into paraffin results in an approximate 66.15% increase in thermal conductivity with the incorporation of 2.0 wt.% graphene [138]. The superior thermal conductivity of these nanomaterials facilitates more efficient heat transfer within the composite, thereby optimizing its thermal properties.

Various treatments can enhance the properties of diatomite, thereby improving its dispersion and interaction with paraffin. For instance, calcining diatomite at temperatures ranging from 450 °C to 650 °C has been shown to enhance its mechanical properties, which can, in turn, positively influence its thermal properties [43]. Additionally, silane coupling agents are frequently employed to augment the interfacial adhesion between diatomite and paraffin, thus enhancing the overall thermal properties of the composite [103]. These modifications can optimize heat exchange performance and bolster the structural integrity of the composite.

The ratio of diatomite to paraffin within the composite is essential for achieving optimal thermal conductivity. An excessive concentration of diatomite may result in diminished thermal performance due to its insulating properties. Research indicates that maintaining an optimal balance, typically around 5–10% by weight of diatomite, can enhance thermal conductivity while preserving the structural advantages conferred by diatomite [139].

Research indicates that the integration of various fillers, such as carbon nanotubes (CNTs) and expanded graphite, in conjunction with diatomite within a paraffin matrix, enhances thermal conductivity synergistically. These hybrid materials not only augment the rate of heat transfer but also preserve other advantageous properties of paraffin as a phase change material (PCM), including its latent heat capacity [140].

Advanced manufacturing techniques, such as in situ polymerization or melt blending during composite production, facilitate a more uniform dispersion of components, which is essential for enhancing thermal properties [141]. Additionally, the application of methods like microemulsion impregnation is recommended to improve thermal properties by optimizing the microstructure of composites [142].

The enhancement of thermal conductivity in paraffin–diatomite composites can be effectively achieved through the strategic incorporation of nanomaterials, appropriate surface modification, optimization of composite ratios, and the utilization of innovative hybrid materials. These methodologies not only enhance thermal energy management but also contribute to a more comprehensive understanding of material behavior under operational conditions. Continued research and development in this field may significantly broaden the applications of paraffin–diatomite composites in energy-efficient technologies.

### 5.3. The Effect of Paraffin Content in the Diatomite Matrix

Research on the thermal and functional characteristics of paraffin-diatomaceous composites demonstrates considerable interest in materials for thermal energy storage, particularly phase change materials (PCM). These composites integrate the high heat capacity of paraffin, a commonly utilized PCM, with the advantageous structural properties of diatomite—a natural siliceous material noted for its high porosity, substantial surface area, and thermal stability [143,144,145].

Diatomite functions as an effective matrix for phase change materials due to its structural properties, which facilitate optimal absorption and retention of paraffin wax. The integration of paraffin into diatomite results in a composite with enhanced thermal properties, particularly regarding latent heat storage and thermal conductivity. Research has demonstrated that paraffin–diatomite composites can achieve a maximum latent heat of approximately 70.51 kJ/kg, indicating substantial heat storage capabilities [101,146]. Moreover, the phase change temperatures of paraffin can be effectively regulated by varying the diatomite content, enabling customized thermal properties suitable for application in building materials and temperature management systems [85,101].

The paraffin content within the diatomite matrix exerts a substantial influence on the thermal and mechanical properties of the resultant composite. An increase in paraffin content enhances the latent heat capacity of the composites; however, it may also diminish mechanical strength due to a reduction in the stiffness of the diatomite phase. Previous studies have demonstrated that augmenting the paraffin content from 30% to 50% correlates with a decrease in compressive strength, suggesting a threshold beyond which mechanical properties may significantly deteriorate [146]. This relationship underscores the necessity for meticulous formulation to optimize thermal properties while preserving mechanical strength.

The functional properties, including thermal stability and moisture resistance, are significantly affected by the interaction between paraffin and diatomite. The inherent porous structure of diatomite facilitates the effective encapsulation of paraffin, thereby preventing leakage during phase transitions and enhancing the composite’s stability. The diatomite matrix’s capacity to form a protective layer around the paraffin wax further contributes to moisture resistance and the overall durability of the composite [101,147]. Consequently, the performance of paraffin–diatomite composites can be optimized by adjusting the proportions to ensure that thermal properties meet the functional requirements of specific applications, such as thermal energy storage systems or insulating materials for buildings [148].

The interaction between paraffin and diatomite is essential in determining the thermal and functional properties of these composites. By adjusting the paraffin content within the diatomite matrix, significant enhancements in thermal properties can be achieved, although challenges related to maintaining mechanical integrity may arise. Consequently, further research into optimizing these composites is essential for maximizing their effectiveness in applications related to thermal energy storage and insulation technologies.

### 5.4. Resistance to Leakage During Melting–Crystallization Cycles

The characteristics of paraffin–diatomite composites, particularly their resistance to leakage during melting and crystallization cycles, are essential for their utilization in thermal energy storage systems. This material combination enhances thermal properties and addresses the challenge of maintaining structural integrity while simultaneously preventing the leakage of the phase change material (PCM) during operation.

Paraffin wax is extensively utilized as a phase change material (PCM) due to its advantageous thermal properties, notably its high heat capacity, which typically ranges from 200 kJ/kg to 300 kJ/kg. Nevertheless, its application may be constrained by leakage during phase transitions from the solid to the liquid state. Diatomite, a naturally occurring siliceous material, possesses a porous structure that effectively encapsulates paraffin wax. By employing diatomite as a matrix, the composite seeks to address issues related to leakage while concurrently enhancing the thermal performance of the PCM [85].

The geophysical characteristics of diatomite play an essential role in influencing the performance of paraffin–diatomite composites. Its high porosity and extensive surface area facilitate the effective absorption and retention of paraffin wax, thereby mitigating the risk of leakage during melting and solidification cycles. This structural encapsulation leads to the formation of SS-PCMs, wherein the paraffin is immobilized, preventing its escape during thermal cycles [42].

Leak resistance in paraffin–diatomite composites is achieved through several mechanisms. The microstructure of diatomite forms a network of capillaries that not only encapsulate the paraffin but also enhance the contact surface area for thermal exchange. This configuration facilitates improved heat transfer while effectively retaining the paraffin during its phase transition. Empirical studies have demonstrated that the porous nature of diatomite contributes to maintaining the solid phase of the composite, thereby counteracting forces that induce leakage at elevated temperatures [147].

Furthermore, the interaction between paraffin and diatomite is characterized by physical forces that enhance strong bonding, thereby further stabilizing the structure. Enhanced interfacial adhesion reduces fluid migration during phase transitions, significantly mitigating the risk of leakage. Studies indicate that leakage rates in such composites can be maintained below 5% over numerous thermal cycles, underscoring their reliability [149].

The experimental results indicate that paraffin–diatomite composites exhibit effective performance under various thermal cycling conditions. The encapsulated paraffin consistently retains latent heat and shows minimal variation in melting temperature, typically ranging from 51–55 °C, even after extensive thermal cycling (e.g., over 200 cycles) [147]. The absence of leakage during these cycles confirms the efficacy of diatomite as a stabilizing agent, resulting in a stable composite capable of enduring repeated phase transitions without compromising material integrity.

Furthermore, enhancements in the design of the composite, such as the incorporation of additives like carbon nanotubes (CNTs) or modified silica into the diatomite structure, can significantly improve its structural properties and thermal transfer characteristics. These materials facilitate the formation of thermal pathways that enhance heat distribution and bolster the composite’s mechanical integrity, thereby reducing its susceptibility to damage that could result in leaks [104].

Paraffin–diatomite composites demonstrate exceptional resistance to leakage during melting and crystallization cycles, rendering them highly suitable for thermal energy storage applications. The structural and thermal benefits of diatomite, coupled with its capacity to encapsulate paraffin, create a system that effectively minimizes leakage risk while enhancing thermal properties. Continued research focused on optimizing composition and processing techniques is expected to further improve the efficacy of these composites, thereby ensuring their long-term applicability in temperature management contexts.

### 5.5. Analysis of Cyclic Durability and Long-Term Stability

The investigation into the cyclic durability and long-term stability of paraffin–diatomite composites is essential for their utilization in thermal energy storage systems. Cyclic durability pertains to the capacity of paraffin–diatomite composites to preserve their thermal properties and structural integrity throughout repeated heating and cooling cycles. Empirical evidence indicates that paraffin–diatomite composites can operate effectively under thermal cycling conditions, with their components maintaining their properties over 1000 to 1200 cycles without significant degradation in performance. This resilience is attributed to the diatomite matrix, which supports the paraffin wax and prevents leakage by encapsulating it within its porous structure [150]. Research demonstrates that innovative composite structures facilitate minimal leakage, typically below 10% over 200 cycles, thereby indicating robust stability and performance in both solid and liquid states [151].

The thermal transitions observed in these composites include a melting temperature of approximately 54.24 °C, accompanied by a slightly lower crystallization temperature, which facilitates efficient energy storage and release [101]. The crystallization process in paraffin–diatomite composites occurs relatively rapidly due to minimal supercooling, thereby enhancing cyclic durability. The incorporation of diatomite contributes to a reduction in the degree of supercooling, resulting in an improved thermal response and expedited resolidification during the cooling phases [152].

Long-term stability pertains to the structural and chemical integrity of paraffin–diatomite composites during extended use. Research suggests that these composites demonstrate favorable thermal stability amidst prolonged temperature fluctuations. The incorporation of diatomite not only enhances thermal stability but also elevates the degradation temperature of the composite. For instance, studies have indicated that the onset temperature of decomposition can increase significantly (e.g., from approximately 580 °C for pure paraffin to about 603 °C for paraffin–diatomite composites) [101]. This enhanced thermal stability is essential for applications involving exposure to high temperatures.

Empirical investigations into these composites have indicated that their mechanical properties remain stable even after extended exposure to thermal cycles. For instance, research has demonstrated that these composites sustain notable heat storage efficiency with minimal mechanical degradation over time. Furthermore, encapsulation in diatomite preserves the structural integrity of paraffin, preventing phase separation or leaks during prolonged use [151].

An essential component of long-term stability is the preservation of thermal properties, which are assessed through differential scanning calorimetry (DSC). The composites exhibited stable thermal properties during both heating and cooling cycles, with only minimal degradation observed between cycles. This indicates that the paraffin retains its phase change efficiency over time [153,154].

The attributes of paraffin–diatomite composites render them outstanding candidates for thermal energy storage applications, owing to their remarkable cyclic durability and long-term stability. The synergy between the high melting point of paraffin and the structural reinforcement provided by diatomite ensures dependable performance during thermal cycles and in scenarios involving extended exposure. Continuous advancements in composite formulations further enhance their robustness, ensuring that paraffin–diatomite composites remain at the forefront of energy-efficient temperature management solutions.

## 6. Potential Applications

**Figure 4:**
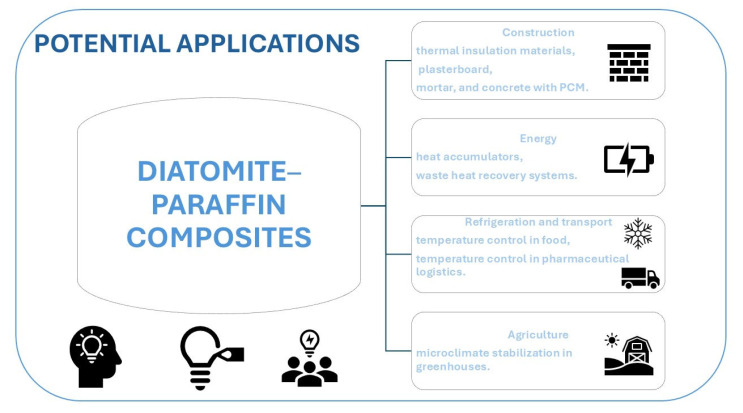
Potential applications of diatomite–paraffin composites in construction, energy, transportation, the refrigeration industry, and agriculture.

Figure 4 illustrates the diverse applications of diatomite–paraffin composites across various economic sectors, highlighting their adaptability and practical importance. In the construction industry, these materials serve as thermal insulation components in gypsum boards, mortars, and concretes containing phase change materials (PCM), facilitating passive indoor temperature regulation and enhancing the energy efficiency of building partitions. Within the energy sector, diatomite–paraffin composites are employed in heat accumulators and waste heat recovery systems, promoting the efficient storage and reuse of thermal energy. In the refrigeration industry and transportation, they are utilized to stabilize temperatures within the food supply chain and pharmaceutical logistics, maintaining consistent thermal conditions without the necessity for a continuous cooling power supply. Lastly, in agriculture, these materials contribute to stabilizing the microclimate in greenhouses, ensuring a more uniform temperature distribution between day and night, thereby fostering plant growth and reducing heating energy consumption.

### 6.1. Construction

The integration of paraffin–diatomite composites into building materials presents a broad spectrum of applications, particularly in the enhancement of thermal insulation, gypsum board products, mortars, and concrete. The distinctive properties of diatomite, when combined with the phase change capabilities of paraffin, result in composites that not only improve thermal properties but also contribute to energy efficiency in construction applications.

Paraffin–diatomite composites demonstrate superior thermal insulation properties due to the synergistic effect of paraffin’s phase-change capability and diatomite’s low thermal conductivity. The porous structure of diatomite effectively entraps air, thereby offering substantial resistance to heat transfer [155]. Research by Costa et al. underscores the potential of these composites to regulate indoor temperatures, a critical factor for achieving energy efficiency in buildings [133].

The integration of paraffin–diatomite composites into gypsum board formulations enhances both thermal and acoustic insulation properties. The heat-retention capabilities of paraffin, coupled with the structural reinforcement offered by diatomite, facilitate improved sound dampening while absorbing thermal energy, thereby contributing to the overall energy efficiency of buildings [101]. Furthermore, research indicates that gypsum boards incorporating diatomite and paraffin can perform effectively across multiple thermal cycles, maintaining both performance parameters and structural integrity [42,156].

The incorporation of paraffin–diatomite composites in mortar formulations constitutes a notable advancement in the domain of building materials, specifically targeting the enhancement of energy efficiency. The substitution of traditional aggregates with diatomite not only augments the mechanical properties of the mortar but also improves thermal insulation. The low density of diatomite facilitates the creation of lighter mixtures, while the inclusion of paraffin enhances the latent heat storage capacity within the mortar matrix [157]. Empirical studies have demonstrated that these composites can effectively diminish thermal conductivity while preserving compressive strength, thereby offering a viable solution for energy-efficient construction applications [158].

Concrete incorporating paraffin–diatomite composites functions as a phase change material (PCM) in construction, demonstrating superior heat storage capabilities. The integration of paraffin enables the concrete to absorb surplus heat during daylight hours and release it nocturnally, thereby facilitating temperature regulation within rooms and diminishing dependence on heating and cooling systems [133,159]. This variant of concrete can be engineered to sustain its performance across diverse climatic conditions and to enhance the energy efficiency of buildings. Empirical evidence indicates that the inclusion of diatomite augments the thermal properties of the composite by ensuring effective distribution and retention of paraffin, thus reducing leakage during phase transitions [101].

Numerous case studies have documented the practical application of paraffin–diatomite composites in real-world construction scenarios. A notable instance is the development of a thermal storage floor employing paraffin/expanded graphite as a composite phase change material (PCM). This system has demonstrated considerable benefits in regulating indoor climate by utilizing the thermal mass of the floor to store surplus heat generated from solar energy, subsequently releasing it during cooler periods. This integration has proven successful in residential heating systems [160]. This approach not only enhances thermal comfort but also exemplifies effective energy efficiency measures in building operations.

A notable application involved the use of geopolymer foams incorporated with diatomite and paraffin granules as thermal insulators in construction. Przybek reported that adjusting the proportions of PCM and diatomite could optimize properties such as thermal conductivity and mechanical strength, thereby producing environmentally sustainable building materials with improved thermal performance [161]. This versatility renders such composites suitable for various building applications, including walls and roofs, where thermal efficiency is paramount.

Furthermore, the research conducted by Zong et al. on stearic acid/diatomite composites delineated methodologies for the preparation of these materials under optimal conditions, thereby significantly enhancing their structural and functional integrity for use in construction [162]. These case studies substantiate the benefits of employing paraffin–diatomite composites in both laboratory and practical applications.

The integration of paraffin and diatomite in the development of composite materials demonstrates significant potential for future applications in the construction industry. Laboratory experiments confirm their thermal efficiency and stability, while practical applications highlight their capacity to enhance energy efficiency and indoor comfort in buildings. Ongoing research and development efforts are essential to further optimize these materials, facilitating their broader adoption in sustainable construction practices.

### 6.2. Energy

Paraffin–diatomite composites exhibit considerable potential in energy applications, particularly in heat storage batteries and waste heat recovery systems. The integration of paraffin as a phase change material (PCM) with diatomite as a carrier matrix enhances the heat storage properties of paraffin, while concurrently augmenting thermal conductivity and structural stability due to the distinctive properties of diatomite.

Paraffin–diatomite composites function effectively as thermal energy storage systems, capable of absorbing, storing, and releasing heat as required. The latent heat storage capacity of paraffin enables it to maintain a stable ambient temperature, thereby facilitating energy conservation. These composites are proficient in regulating indoor climates within both residential and commercial buildings by stabilizing temperature variations and diminishing reliance on heating and cooling systems [101,104].

The thermal conductivity of paraffin–diatomite composites can be enhanced through the incorporation of additives such as carbon nanotubes or graphite, which markedly improve heat transfer efficiency. Research suggests that the direct integration of carbon nanotubes into the paraffin–diatomite matrix results in a significant increase in thermal conductivity without adversely affecting the material’s thermal properties, thereby augmenting the overall performance of thermal energy storage systems [65,163]. The capacity of these composites to function effectively across a range of temperatures expands their applicability in solar energy systems, where efficient heat accumulation is essential for optimizing performance [164]. However, further investigation is necessary to assess the composite’s efficacy in converting solar energy efficiently [163].

Paraffin–diatomite composites in waste heat recovery systems effectively capture surplus thermal energy emitted during industrial processes or energy generation systems. Their elevated melting point facilitates the efficient absorption of waste heat, particularly in scenarios where temperature regulation is critical [165,166].

The integration of paraffin–diatomite composites into waste heat recovery systems offers dual advantages: enhancing thermal energy storage and optimizing energy recovery. The high porosity of diatomite not only aids in the retention of paraffin but also facilitates efficient heat transfer from the waste source to the phase change material (PCM), thereby improving recovery rates [101,102].

Furthermore, due to effective encapsulation, paraffin–diatomite composites are capable of minimizing leakage during phase transitions, thereby preserving their thermal properties even under prolonged cycling conditions. This characteristic ensures the reliable recovery of stored energy when required [48,167]. The incorporation of diatomite mitigates the supercooling effect, which can impede the thermal discharge process, while also facilitating effective crystallization. Consequently, these composites emerge as viable candidates for long-term energy storage applications, particularly in waste heat recovery [48].

The integration of paraffin–diatomite composites into thermal regulation systems has demonstrated promising outcomes in both laboratory experiments and practical applications, particularly in diminishing the cooling load of buildings. This synthesis of experimental results with practical implementations highlights the potential of these materials to enhance energy efficiency in thermal management.

In practical applications, the incorporation of paraffin–diatomite composites in construction materials has resulted in notable reductions in cooling loads. A case study conducted by Ghetany et al. demonstrated that buildings utilizing PCM solutions, including paraffin–diatomite composites, achieved up to a 19.2% reduction in indoor air temperature during peak hours, thereby effectively decreasing the demand for cooling energy [168]. This finding suggests that the phase change properties of these materials directly enhance the building’s energy efficiency, leading to a diminished reliance on conventional HVAC systems.

Another pertinent application involved the incorporation of paraffin–diatomite composites into wall construction. Simulations demonstrated that these materials could substantially decrease cooling demands. For example, Lamastra et al. reported a 10% reduction in the required cooling power in simulated building envelopes treated with phase change materials, including those based on diatomite [169]. This reduction highlights the efficacy of these composites in enhancing thermal mass and stability.

Furthermore, the research conducted by Shi et al. highlighted the efficacy of phase change material (PCM) boards composed of paraffin–diatomite composites, which were applied in a full-scale office setting. The study demonstrated a significant reduction in energy consumption, with a decrease of 18.8% during the summer and 19.7% in the winter, thereby illustrating the practical advantages of employing these materials in thermal storage flooring [170]. The consistent performance across different seasons further underscores the robust thermal regulation capabilities of paraffin–diatomite composites.

The incorporation of paraffin–diatomite composites into thermal regulation systems constitutes a promising area of study, merging laboratory research with practical applications. Empirical tests have verified the high latent heat capacity, stability, and thermal conductivity of these composites, while practical implementations have demonstrated their efficacy in substantially reducing building cooling loads. Consequently, further research and development are necessary to optimize and standardize these materials for broader application in sustainable construction practices.

### 6.3. Refrigeration Industry and Transport

Paraffin–diatomite composites are of considerable importance in the refrigeration and transportation sectors, particularly for temperature regulation in the logistics of food and pharmaceuticals. The integration of paraffin as a phase change material (PCM) with diatomite as a structural support provides distinct advantages, including effective temperature management and stability throughout phase change cycles.

In the domain of food logistics, the maintenance of appropriate temperature conditions during transportation is essential for preserving both the safety and quality of food products. Paraffin–diatomite composites serve as effective thermal packaging or insulating materials, primarily due to their capacity to store latent heat. These composites are capable of absorbing excess heat during transit, thereby mitigating temperature fluctuations that could potentially result in product spoilage. The efficacy of temperature regulation is further augmented by the high specific surface area of diatomite, which facilitates the efficient distribution of paraffin within the composite matrix [45].

Furthermore, the integration of paraffin with diatomite facilitates the development of lightweight, thermally efficient packaging capable of sustaining low temperatures over prolonged durations. The thermal characteristics of these composites have been empirically validated, demonstrating their ability to satisfy the requirements of various food products [102]. This application is particularly advantageous for perishable goods necessitating stringent temperature regulation from distribution centers to retail outlets.

The pharmaceutical industry derives considerable advantages from the application of paraffin–diatomite composites. Numerous pharmaceutical products necessitate stringent temperature regulation to preserve their efficacy and stability. Composites composed of paraffin and diatomite can be engineered to ensure stable temperature management within transportation systems. For instance, the stable incorporation of paraffin within diatomite matrices reduces the risk of leakage during temperature fluctuations, rendering these composites particularly suitable for the transportation of sensitive pharmaceutical cargo [42].

Research demonstrates that these composites consistently preserve their thermal properties across multiple cycles of melting and crystallization, a critical attribute for the repeated heating and cooling processes encountered in logistics [102]. The adaptability of paraffin–diatomite composites enables their customization to meet the diverse temperature thresholds required by various pharmaceutical products. Their capacity to maintain efficacy over numerous thermal cycles ensures that temperature-sensitive transported products remain within acceptable limits.

The thermal conductivity of paraffin–diatomite composites can be further enhanced by incorporating nanofillers such as carbon nanotubes, which have been shown to significantly improve heat transfer rates [104]. This enhancement enables these composites not only to store thermal energy more effectively but also to respond rapidly to fluctuations in ambient temperature, thereby augmenting the overall efficiency of temperature control systems in logistics. Consequently, cargo transportation can be conducted more efficiently, leading to reduced energy consumption and costs associated with refrigeration systems.

Paraffin–diatomite composites demonstrate significant efficacy in temperature regulation for the logistics of food and pharmaceuticals. Their attributes, such as proficient heat energy absorption, stability during phase transitions, and the potential to enhance thermal conductivity through the incorporation of additives, render them suitable for preserving the integrity of temperature-sensitive cargo. As the logistics sector increasingly prioritizes energy efficiency and sustainability, these composites offer a promising solution for maintaining optimal storage conditions throughout the entire transportation process.

### 6.4. Agriculture

The utilization of paraffin–diatomite composites in agriculture, particularly for stabilizing the microclimate within greenhouses, presents a promising approach to enhancing plant growth and energy efficiency. These composites exploit the thermal properties of paraffin, which serves as a phase change material (PCM), in combination with the structural advantages of diatomite, to effectively regulate temperature fluctuations in the greenhouse environment.

Paraffin–diatomite composites demonstrate exceptional thermal energy storage capabilities, enabling them to absorb heat during daylight hours and release it gradually during nighttime. This thermal buffering capacity is essential for maintaining stable temperatures within greenhouses, which is vital for optimal plant growth [171]. Research suggests that integrating these composites into greenhouse structures can mitigate temperature fluctuations, thereby creating a more controlled environment for crops both during the day and at night [172].

Furthermore, the capacity of paraffin to undergo phase transitions at specific temperatures is particularly beneficial. When integrated with diatomite, this composite can significantly enhance the storage and release of thermal energy, thereby preventing plants from being subjected to detrimental extreme temperatures [173]. By stabilizing the internal microclimate, these composites can facilitate an extended growing season and enhance overall agricultural yields. Empirical studies have demonstrated that improved thermal insulation can result in substantial energy savings and increased yields in greenhouse environments [174].

The integration of diatomite with paraffin significantly enhances the energy efficiency of greenhouses due to their insulating properties. The low thermal conductivity of diatomite, coupled with the latent heat capacity of paraffin, forms an effective barrier against heat loss, thereby reducing the necessity for supplementary heating systems during colder periods [42]. This insulating capability is particularly essential in regions experiencing frequent temperature fluctuations, facilitating more energy-efficient greenhouse operations [171].

Furthermore, research highlights that the incorporation of paraffin–diatomite composites can substantially decrease energy consumption for heating by employing efficient thermal screens. These screens can reduce heat loss through greenhouse coverings by up to 60% during nighttime [172,174]. This aspect is particularly significant in agricultural practices focused on sustainable development, as diminishing energy consumption is directly associated with a reduction in greenhouse gas emissions related to heating and cooling.

The integration of paraffin–diatomite composites into greenhouse designs can be accomplished through various approaches, such as incorporating them into the greenhouse cladding or employing them as thermal materials in construction [173]. Their lightweight properties facilitate easier handling and installation compared to conventional insulating materials [175]. Moreover, research on multilayer thermal screens suggests that optimizing the composition and layering of these composites can enhance thermal regulation, offering multifunctional advantages, including humidity control and condensation management within the greenhouse environment [104,176].

Paraffin–diatomite composites represent a notable advancement in the stabilization of greenhouse microclimates. Due to their effective temperature regulation and enhanced energy efficiency, these materials hold the potential to support sustainable agricultural practices. As the agricultural sector increasingly endeavors to mitigate the impacts of climate change and enhance productivity, the integration of such innovative materials is likely to become indispensable in future farm management strategies.

### 6.5. Correlation Between Performance Requirements and Material Optimization Strategies

The efficacy of paraffin–diatomite composite phase change materials (PCMs) is significantly influenced by the interaction between their functional requirements—such as thermal conductivity, latent-heat retention, cyclic stability, mechanical integrity, and cost-effectiveness—and the material optimization strategies employed during synthesis. These strategies include surface modification, the incorporation of high-conductivity fillers, impregnation control, matrix selection, and processing techniques [177,178,179,180].

*(a)*  *Thermal conductivity vs. structural optimization*

Enhancing effective thermal conductivity represents a pivotal aspect of phase change material (PCM) optimization. The incorporation of carbon-based materials, such as graphene, carbon nanotubes (CNTs), or metallic nanoparticles, has been shown to enhance thermal conductivity by 40–70% while preserving over 90% of the latent heat capacity [181,182,183,184,185]. Nevertheless, an excessive filler content exceeding 3 wt.% may diminish PCM loading efficiency and enthalpy [186]. Consequently, it is imperative to balance filler concentration and dispersion uniformity to sustain high heat transfer without compromising phase-change efficiency [187].

*(b)*  *Cyclic stability vs. surface modification of the support*

The long-term cyclic reliability of phase change materials (PCMs)—defined as their capacity to maintain latent heat and prevent leakage during repeated melting–solidification cycles—is significantly influenced by the surface chemistry of the support material. Chemical modifications of diatomite, such as silanization and calcination, improve the interfacial bonding between paraffin and the silica framework, thereby reducing leakage to below 5% after 500 cycles [169,188,189]. Additionally, the application of polymeric coatings and hybrid systems incorporating nanofillers further mitigates degradation, preserving over 90% of the initial enthalpy [190,191].

*(c)*  *Mechanical strength vs. matrix composition*

In structural and building applications, it is essential to achieve a balance between thermal efficiency and mechanical strength. The incorporation of paraffin typically reduces compressive strength due to the softness and volume change in the PCM phase. The addition of diatomite or other porous minerals mitigates this effect by providing stiffness and interfacial adhesion [161]. The use of geopolymer or lightweight mineral binders further enhances structural integrity while maintaining adequate heat-storage capacity [192]. Consequently, a clear correlation exists between matrix selection and the thermo-mechanical balance of PCM composites [193,194].

*(d)*  *Cost-effectiveness vs. process scalability*

Simple techniques such as vacuum impregnation or capillary adsorption remain appealing for large-scale production due to their cost-effectiveness and scalability, despite offering moderate conductivity enhancement (10–25%) [117]. In contrast, more advanced chemical and hybrid modification methods (e.g., CNT/graphene, metal oxides) provide superior thermal performance but entail higher energy and equipment costs [195]. Consequently, optimizing the cost–benefit ratio necessitates balancing processing complexity with achievable performance, particularly in the context of energy-efficient building applications [196].

**Figure 5:**
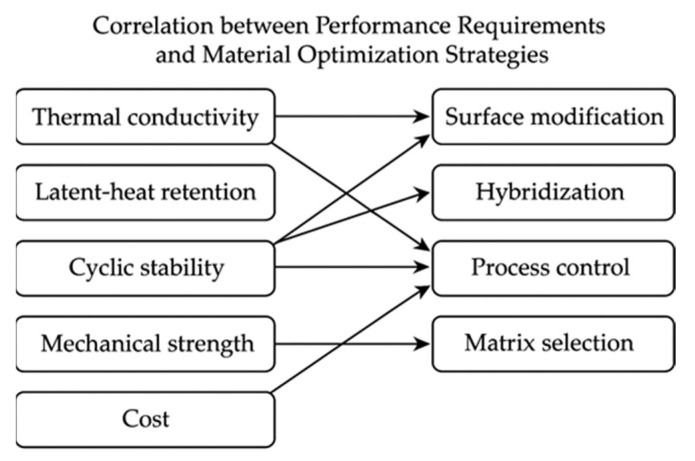
The major correlations linking performance requirements and optimization strategies for paraffin-based composite phase-change materials.

A schematic analysis (Figure 5) indicates that the most significant correlations are observed between thermal conductivity and cyclic stability, as these parameters exert the greatest influence on the overall efficiency of PCM systems [177,181,182,186,188]. To maintain mechanical strength and cost-effectiveness, it is necessary to employ hybrid strategies that integrate moderate surface modification with controlled filler addition [161,169,189,195].

## 7. Comparison with Other PCM Encapsulated Porous Materials

The properties and performance of paraffin–diatomite composites have been systematically evaluated in comparison to other commonly utilized phase change materials (PCMs), including those based on perlite, zeolite, and silica.

In the context of thermal properties, paraffin–diatomite composites demonstrate a latent heat capacity of approximately 61.96 kJ/kg [101], which surpasses that of perlite-based composites, approximately 57.30 kJ/kg [104], and zeolite-paraffin composites, which typically range from 50 to 55 kJ/kg, contingent upon the specific composition [197]. Silica-based phase change materials (PCMs) exhibit a latent heat capacity of approximately 55.20 kJ/kg [198]. The thermal conductivity of paraffin–diatomite composites spans from 0.5 to 0.8 W/m·K [101], markedly exceeding that of expanded perlite composites (0.06–0.10 W/m·K [109]), zeolite-based composites (0.1–0.3 W/m·K, contingent on moisture content; [133]), and silica-enhanced PCMs (0.15–0.4 W/m·K [48]), thereby indicating an enhanced heat transfer capability.

Paraffin–diatomite composites exhibit superior structural integrity and thermal reliability, demonstrating excellent resistance to leakage, with no significant paraffin release observed over 200 thermal cycles [101]. Conversely, perlite-based composites show minor leakage after approximately 150 cycles, attributed to volumetric changes associated with expansion and compression [104]. Zeolite-based systems exhibit variable leakage behavior due to moisture absorption, which can adversely affect cycle stability [197]. Although silica matrices generally maintain stability, they may experience limited leakage during extended thermal cycling due to incomplete hermetic sealing [198].

The efficacy of paraffin–diatomite composites has been assessed in the context of energy storage and construction applications, revealing a notable reduction in annual heating energy consumption, with experimental investigations indicating energy savings of up to 40% [101]. In contrast, perlite-based phase change materials (PCMs) employed in agricultural settings exhibited a diminished capacity to stabilize thermal conditions, with a performance decrease of 15–20% compared to diatomite composites [109]. Zeolite-based PCMs generally demonstrate effectiveness; however, their performance can decline by as much as 30% due to humidity fluctuations in controlled agricultural environments [133]. Silica composites are capable of achieving approximately 25% energy savings in passive building applications, although they necessitate a higher energy input for activation [48].

In terms of thermal stability during cyclic operation, paraffin–diatomite composites exhibit minimal temperature fluctuations of ±2 °C across repeated cycles [101]. In contrast, perlite-based systems demonstrate fluctuations of ±4 °C [40], while zeolite-based composites frequently exceed ±5 °C, contingent upon environmental conditions [197]. Silica-based phase change materials (PCMs) generally display fluctuations within ±3 °C, although their performance is significantly influenced by the material composition [198].

The data highlight the superior thermal performance, enhanced structural stability, and improved practical applicability of paraffin–diatomite composites compared to other phase change material (PCM) systems. This indicates their suitability for applications in energy-efficient building materials, thermal energy storage, and temperature-sensitive logistics.

Diatomite has emerged as a favored material in the development of composites with phase change materials (PCM) due to its distinctive properties, including high porosity, extensive surface area, and exceptional thermal stability. Nevertheless, it is essential to evaluate the advantages and disadvantages of diatomite in comparison to alternative materials in PCM composites, such as perlite, zeolite, and silica. This evaluation underscores the potential contributions of each of these materials in enhancing applications related to thermal energy storage.

The incorporation of diatomite into phase change material (PCM) composites offers several advantages, notably its high porosity and specific surface area. Diatomite is distinguished by its exceptional porosity, which facilitates the absorption and storage of PCMs, thereby significantly enhancing its sorption capacity and enabling effective energy storage [45,199]. In comparison to perlite and zeolite, diatomite frequently demonstrates superior liquid absorption and retention capabilities, which are essential for preventing leaks [194]. A further notable advantage of diatomite is its thermal stability, which allows it to endure high temperatures without degradation. Research indicates that the thermal conductivity of diatomite generally ranges from 0.07 to 0.4 W/(m·K), a moderate value relative to other materials employed in PCM production [199]. This stability ensures that diatomite-based composites perform effectively over numerous thermal cycles, rendering them ideal for applications necessitating durability [45]. Moreover, diatomite is relatively inexpensive and widely available, making it an attractive option for large-scale applications. Its natural abundance reduces production costs compared to synthetic materials such as silica aerogels or engineered zeolites, which may offer superior properties but at a higher cost [200]. Diatomite also effectively regulates moisture levels due to its large surface area and hydrophilic properties. This characteristic is particularly advantageous in agricultural applications or environments requiring moisture management, where excessive humidity can negatively impact crop health or the integrity of materials [105,201].

Among the limitations of diatomite in phase change material (PCM) composites, its moderate thermal conductivity warrants attention. While diatomite exhibits satisfactory thermal conductivity, it generally falls short when compared to conventional materials such as graphite or certain metal composites. Consequently, although diatomite-based PCMs are beneficial for heat storage, they may necessitate the incorporation of supplementary materials to enhance heat transfer during the charging and discharging phases [202]. Furthermore, the mechanical properties of diatomite-based composites do not consistently match those of alternative materials, such as silica or specially engineered polymers. Some studies suggest that while diatomite can improve certain properties, it may also result in diminished compressive strength when it replaces traditional building materials in composites [203]. This limitation may require additional reinforcement to preserve structural integrity, particularly in applications where load-bearing capacity is critical [201]. Additionally, the use of diatomite may induce supercooling in some PCM applications, a phenomenon that leads to thermal inefficiency by preventing the PCM from solidifying or melting at the anticipated temperatures, thereby diminishing performance in temperature-sensitive systems [42,104]. The extraction and processing of diatomite can also have environmental repercussions, akin to other mining operations. Although generally considered benign, increased extraction from natural sources may raise concerns regarding long-term sustainability [200,201].

Perlite is characterized by its lightweight nature and excellent insulating properties; however, it lacks the absorbency of diatomite. Furthermore, the thermal conductivity of perlite is generally lower than that of diatomite-based composites, which restricts its application in contexts requiring efficient heat transfer and rapid response times [204]. Zeolite, on the other hand, possesses moisture adsorption and superior ion-exchange properties, rendering it advantageous in certain applications. Nevertheless, zeolite may exhibit lower thermal stability compared to diatomite, potentially leading to diminished performance at elevated temperatures [205,206]. Silica offers high thermal performance and strength, yet it is typically associated with higher costs and is less sustainable than diatomite. The energy-intensive process of synthesizing silica may not align with sustainability objectives for composites derived from natural materials such as diatomite [45,201]. Diatomite presents several advantages as a PCM composite carrier, including high porosity, thermal stability, and cost-effectiveness, though it also has limitations, such as moderate thermal conductivity and potential mechanical constraints. In comparison to alternative materials like perlite, zeolite, and silica, diatomite can provide significant benefits in specific applications, particularly in thermal energy storage systems. The selection among these materials must consider the specific requirements of the application, including temperature management, structural needs, and environmental sustainability.

## 8. Challenges and Directions of Development

### 8.1. Increase in Thermal Conductivity

The advancement of paraffin–diatomite composites, particularly in enhancing their thermal conductivity through the incorporation of nanofillers such as graphene, carbon nanotubes (CNT), and metal particles, has garnered considerable attention from the research community. This innovation is driven by the need for more efficient thermal energy storage materials that maintain high latent heat while addressing the inherent low thermal conductivity of paraffin-based composites.

Research has demonstrated that the integration of carbon nanotubes into paraffin–diatomite composites markedly enhances thermal conductivity. Specifically, multi-walled carbon nanotubes (MWCNT) have been shown to significantly improve the heat exchange efficiency in phase change materials (PCM) due to their high thermal conductivity. Empirical findings suggest that certain configurations can achieve substantial improvements without adversely affecting other thermal properties [138,207]. A composite containing 0.26 wt.% MWCNT exhibited a 42.45% increase in thermal conductivity, underscoring the efficacy of carbon nanotubes as additives in these systems [137,207]. These enhancements are attributed to improved thermal contact between the paraffin and nanoadditives, facilitating efficient heat transfer [208].

Furthermore, the incorporation of metal particles, such as aluminum, has demonstrated significant advantages. Research indicates that the inclusion of aluminum powder not only enhances thermal conductivity but also synergistically improves the overall heat storage capacity of paraffin composites [209]. Of particular interest is the latent heat storage capacity of paraffin–diatomite composites, with specific combinations of paraffin and diatomite achieving values of approximately 61.96 kJ/kg [101].

Graphene derivatives constitute a significant category of nanoadditives that enhance thermal properties. The incorporation of graphene oxide is linked to improved thermal conductivity, attributable to its distinctive structural characteristics that promote more efficient heat transfer within the paraffin matrix [210]. Recent research indicates that a paraffin-graphene composite can achieve an increase in thermal conductivity of up to 37.1%, contingent upon the concentration of graphene nanoplatelets [144].

Furthermore, boron nitride nanolayers (BNNS) were incorporated into the composites to achieve remarkably high thermal conductivity, exceeding the values observed in pure paraffin. The thermal conductivity of composites containing BNNS can reach up to 30.6 W/m·K, representing a significant advancement in addressing the thermal limitations associated with conventional paraffin materials [211].

The integration of nanoadditives, including carbon nanotubes, graphene, and metals, into paraffin–diatomite composites constitutes a promising strategy for enhancing thermal conductivity and broadening their potential applications in energy storage systems. Further investigation into the optimal types and concentrations of these additives may further augment the thermal properties of these materials, ultimately contributing to more efficient energy management solutions.

### 8.2. Improvement of Synthesis Methods for Greater Control over Paraffin Distribution in the Matrix

The synthesis of paraffin–diatomite composites represents an emerging area within materials science, particularly concerning the methodologies employed to optimize the distribution of paraffin within the diatomite matrix. These composites are engineered to enhance thermal properties while preserving structural integrity, making them suitable for application in thermal energy storage (TES) systems.

A primary challenge in the synthesis of effective paraffin–diatomite composites lies in achieving a uniform distribution of paraffin within the composite matrix. Inadequate dispersion can result in agglomeration and inconsistent thermal properties, which directly impact the composite’s performance. Research indicates that the method of paraffin incorporation is essential. Techniques such as direct impregnation, wherein paraffin is introduced into the porous structure of diatomite, have been extensively investigated. Nevertheless, this method frequently leads to uneven filling due to the highly porous nature of diatomite, necessitating precise control of processing conditions to enhance overall dispersion and minimize the risk of paraffin leakage [38,102].

An alternative approach involves employing hydrothermal or in situ polymerization techniques, which enable more effective regulation of the interaction between diatomite and paraffin. For instance, in situ polymerization can enhance the integration of paraffin with the diatomite walls, resulting in a more stable composite that reduces leakage during phase change cycles. However, among the candidates presented, no specific references confirming in situ polymerization in this context were identified, necessitating caution when formulating claims [146].

Recent advancements have demonstrated that altering the surface chemistry of diatomite can enhance its interaction with paraffin, thereby increasing the overall stability of the composite. For instance, surface treatment employing functional amines or other coupling agents can establish a more favorable bonding environment, effectively anchoring the paraffin and improving its dispersion throughout the matrix [212,213]. These modifications address the challenge of achieving uniform thermal conductivity across the entire composite, ensuring efficient heat transfer during operation.

The intrinsic properties of diatomite, particularly its porosity and surface area, are essential in determining the efficacy of the synthesis method. The extent of diatomite porosity permits varying paraffin loads; however, it necessitates careful regulation to prevent excessive diatomite content, which could result in undesirable mechanical properties such as increased brittleness and diminished structural integrity during thermal cycling [139]. Consequently, optimizing the paraffin-to-diatomite ratio and the characteristics of the diatomaceous earth employed is imperative for the development of high-performance composites for thermal energy storage.

Future research on the development of paraffin–diatomite composites should prioritize the enhancement of synthesis methods to improve the physical interactions between paraffin and diatomite. Employing strategies such as advanced surface modifications, controlled thermal processing techniques, and precise material formulations can substantially contribute to achieving superior performance indicators for these innovative materials in thermal energy applications.

### 8.3. Analysis of the Impact of Operating Conditions (Humidity, Aging, Contact with Other Materials)

The operational conditions exert a substantial influence on the properties and performance of paraffin–diatomite composites. Critical factors, including humidity, aging, and interaction with other materials, can significantly affect the efficacy of these composites in applications pertaining to thermal energy storage.

Moisture constitutes a primary concern due to its impact on the stability of both paraffin and diatomite. The presence of moisture can induce hydrolysis reactions or other detrimental effects that compromise the structural integrity of diatomite, which functions as the matrix for the paraffin phase change material (PCM). Empirical studies have demonstrated that elevated humidity levels can cause diatomite to swell, potentially leading to the degradation of mechanical properties and premature damage to the composite [42]. Additionally, moisture can influence the sorption capacity of diatomite, resulting in alterations to the latent heat of the composite, as water interferes with the phase change mechanisms of paraffin [45]. For instance, Ramakrishnan et al. reported that the interaction between water and paraffin during mixing processes can result in significant PCM leakage, thereby constraining the performance of the composite [214].

Aging constitutes a further substantial challenge. Over time, paraffin is subject to thermal cycles, resulting in thermal expansion and contraction, which may impose stress on the diatomite structure, potentially leading to microcracks or other forms of degradation that compromise the long-term efficacy of the composite [147]. Research has demonstrated that the continuous aging of paraffin–diatomite composites can impact their mechanical and thermal properties, ultimately diminishing latent heat storage capacity and performance [215]. Additionally, the chemical stability of paraffin within the diatomite matrix may be adversely influenced by prolonged exposure to elevated or fluctuating temperatures [45].

Interactions with other materials significantly influence the properties of paraffin–diatomite composites. The introduction of additives or other substances during the synthesis phase can lead to competitive chemical reactions that alter the overall properties of the composites [207]. For instance, the addition of certain salts or hydrophilic materials can enhance the compatibility of paraffin with diatomite, thereby improving thermal conductivity and storage capacity. Conversely, some additives may induce phase separation or other issues that impede the desired phase change properties [102]. Research conducted by Fu et al. underscores the importance of optimal material selection and integration techniques in achieving stable and efficient phase change systems [147].

Furthermore, when phase change materials (PCMs) are integrated into cementitious composites, particularly within thermal energy storage systems, improper handling can result in substantial defects due to interactions with water and the chemical constituents of the cement, thereby elevating the risk of PCM leakage [214]. The equilibrium of such additives, along with the water content during the mixing and curing processes, significantly influences the ultimate physical properties of the composite material.

Comprehending the influence of environmental and operational conditions on the behavior and performance of paraffin–diatomite composites is essential for advancing their applications in thermal energy storage. To address these challenges and enhance the reliability of these innovative materials, it is imperative to further refine synthesis methods and environmental management strategies.

### 8.4. Economic and Ecological Assessment of PCM Production with Diatomite

The advancement of paraffin–diatomite composites as phase change materials (PCM) for thermal energy storage necessitates a thorough economic and environmental evaluation of the related production processes. To enhance sustainable development, it is imperative to consider factors such as the costs of raw materials, processing efficiency, waste generation, and potential environmental impacts.

The economic feasibility of producing paraffin–diatomite composites is contingent upon the cost-effectiveness of raw materials and the synthesis methods employed. Diatomite, as a naturally occurring material, offers a cost-effective alternative to synthetic materials. Nonetheless, the economic analysis must also consider the functional advantages derived from its application as a carrier material for paraffin. Various studies suggest that the optimal diatomite content is critical; exceeding 10% by mass may result in agglomeration and inadequate dispersion within the matrix, thereby escalating production costs due to inefficiency [99,139]. Consequently, economic modeling should prioritize identifying the ideal diatomite concentration that optimizes performance while minimizing costs.

Furthermore, production techniques such as vacuum impregnation and various modification methods significantly impact the functional properties of composites and influence their overall economic feasibility. Processes that are energy-intensive or require costly equipment can substantially elevate the final product’s cost. Composite preparation methods, including vacuum impregnation, entail higher initial and operational expenses but can yield superior composite quality [99,101]. Consequently, achieving a balance between operational costs and production quality is essential for sustainable economic development.

From an environmental protection standpoint, the production of paraffin–diatomite composites necessitates a comprehensive evaluation of the impacts associated with the extraction, processing, and eventual disposal of materials throughout the product’s life cycle. While the extraction of diatomite is associated with relatively minor environmental impacts, it nonetheless requires meticulous management to mitigate landscape disruption and preserve ecological balance [39]. Furthermore, initiatives aimed at utilizing diatomite waste from industrial processes, particularly those related to palm oil production, offer a promising strategy for enhancing sustainability by minimizing waste and reducing the demand for primary raw materials [39,197].

In the synthesis of these composites, it is imperative to account for the consumption of chemicals and the potential generation of by-products. Numerous processes associated with the modification of diatomite or the preparation of paraffin–diatomite composites may produce waste or emissions that adversely impact air and water quality. Advancements in green chemistry, such as the implementation of non-toxic solvents or biodegradable additives, can mitigate the environmental impact of production [212]. Additionally, regulations of waste disposal and emission standards further shape the operational decisions available to manufacturers.

Future research endeavors should prioritize enhancing the sustainability of the entire life cycle of paraffin–diatomite composites. This may involve identifying renewable sources of paraffin, optimizing production techniques to minimize energy consumption, and devising methods for recycling composite materials at the end of their life cycle. Additionally, to achieve a comprehensive understanding of the environmental impact of these materials, it is imperative to conduct studies that assess their effects in real-world applications [199].

An integrated economic and environmental assessment is essential in the development of paraffin–diatomite composite production. This approach ensures that these materials are not only effective for thermal energy storage but also sustainable and economically viable throughout their entire life cycle.

## 9. Summary and Prospects

### 9.1. Main Conclusions Regarding the Advantages and Limitations of Paraffin–Diatomite Composites

The evaluation of paraffin–diatomite composites necessitates a thorough assessment of their advantages and limitations to ascertain their efficacy as phase change materials (PCM) in thermal energy storage applications.

Paraffin–diatomite composites are distinguished by their substantial heat capacity, with empirical studies indicating that these composites can achieve latent heat values as high as 87.09 kJ/kg for specific formulations [104]. This characteristic renders them highly suitable for diverse temperature management applications, particularly in building materials where thermal performance is critical. The structural integrity of paraffin is notably enhanced when integrated with diatomite, as diatomite contributes to the stabilization of paraffin’s shape during phase transitions, thereby mitigating leakage and phase separation. However, it is important to note that no specific studies have been identified that explicitly confirm this stability; thus, this assertion, while generally accepted, should be considered with caution [111]. Diatomite, being a naturally abundant material, offers an environmentally sustainable alternative to purely synthetic fillers in phase change materials (PCM). The incorporation of diatomite not only facilitates the utilization of renewable resources but also enhances the mechanical properties of the composite without significantly increasing its weight [103]. Furthermore, the processing of diatomite waste enhances its applicability while reducing the environmental impact associated with disposal [216]. The inclusion of diatomite can augment the thermal conductivity of paraffin-based composites, thereby facilitating more efficient heat transfer. This effect is particularly pronounced when supplemented with nano-additives such as carbon nanotubes, which further optimize thermal properties [104]. The porous structure of diatomite enables improved absorption and redistribution of heat, which is essential in heat storage applications [45].

A notable limitation is the challenge of achieving uniform dispersion of paraffin within the diatomite matrix. An excessive content of diatomite can result in agglomeration, which compromises the mechanical and thermal properties of the composite. Research indicates that maintaining the diatomite dosage below 10% by mass is essential to prevent these negative effects and to optimize properties such as creep resistance [139]. The hydrophilic nature of diatomite can lead to moisture absorption, potentially impairing thermal performance and increasing the risk of PCM leakage during heating and cooling cycles. Addressing moisture sensitivity may require further modifications or the application of protective coatings [217]. Although paraffin–diatomite composites exhibit satisfactory performance during thermal cycling, their long-term reliability necessitates careful evaluation. Some studies suggest that continuous exposure to temperature fluctuations may impact mechanical and thermal stability, yet the specific mechanisms of degradation in diatomite-enhanced composites warrant further investigation [218]. While diatomite is an inexpensive natural raw material, the processing and treatment required to enhance its compatibility and performance with paraffin may elevate production costs. Additionally, advanced processing techniques such as vacuum impregnation, which entail higher operational costs, may restrict broader commercial application [101].

Paraffin–diatomite composites offer a promising avenue for enhancing thermal energy storage, attributable to their substantial heat capacity, stability, and properties that align with sustainable development goals. Nonetheless, to optimize their applicability in practical scenarios, challenges related to dispersion, moisture sensitivity, stability during thermal cycles, and cost must be addressed. Future research should focus on refining processing techniques and improving the interaction between paraffin and diatomite to fully harness the potential of these composites in energy-efficient systems.

Paraffin–diatomite composite phase change materials (PCMs) offer several notable advantages, including a high latent-heat capacity, effective leakage prevention due to porous confinement, chemical inertness, and compatibility with various matrices such as polymers, geopolymers, and cementitious materials. These attributes render them promising candidates for applications in building energy management, thermal regulation, and renewable energy storage systems [45,112,161]. Nevertheless, certain limitations continue to impede their large-scale implementation. These limitations encompass non-uniform paraffin dispersion, poor intrinsic thermal conductivity, and relatively high production costs associated with surface modification or the use of hybrid fillers such as graphene, carbon nanotubes (CNTs), and metals [120,219,220,221,222]. Furthermore, the trade-off between thermal efficiency and material stability remains a significant challenge, as enhancements in conductivity often compromise PCM encapsulation efficiency or latent-heat retention [122,223,224].

Importantly, the current literature reveals several research contradictions and unresolved issues in this field:

▪ **Thermal conductivity vs. latent-heat capacity**—Some studies report that adding carbon nanomaterials significantly enhances heat transfer, while others show negligible or even negative effects due to filler agglomeration and reduced PCM content [115,225,226].
▪ **Surface modification vs. long-term stability**—Although silanization improves bonding and reduces leakage, repeated thermal cycling can degrade functional groups, leading to gradual loss of stability [227].
▪ **Porosity vs. loading efficiency**—High-porosity diatomite improves PCM encapsulation but simultaneously weakens mechanical strength and structural integrity of composites [187].
▪ **Economic feasibility vs. material performance**—Chemically modified or hybrid composites show outstanding laboratory performance but remain too costly or complex for mass-scale use [184,228].

In conclusion, it is imperative that future research prioritizes multi-objective optimization, encompassing microstructural design, process scalability, and the balance between cost and performance. Hybrid strategies that integrate moderate surface modification with low concentrations of conductive additives show significant promise in achieving both high thermal efficiency and long-term reliability. Additionally, there is an increasing demand for standardized testing protocols to assess cyclic stability and heat-transfer behavior under actual operating conditions, thereby ensuring comparability across studies. As delineated in Table 4, the primary research contradictions in paraffin–diatomite composite PCMs stem from the inherent trade-offs between key performance parameters and material design strategies. The table identifies four predominant areas of conflict: the equilibrium between thermal conductivity and latent-heat retention, the degradation of surface modification layers during cyclic operation, the competition between porosity and mechanical integrity, and the challenge of achieving cost efficiency alongside high performance. Each of these contradictions is accompanied by a proposed optimization direction, such as employing surface-functionalized nanofillers to prevent agglomeration, developing thermally stable coupling agents to enhance cycling durability, or adjusting pore-size distribution to improve structural strength. Table 4 offers a succinct visual summary of the key performance–optimization relationships discussed in this section and outlines practical pathways for enhancing the long-term stability, scalability, and cost-effectiveness of paraffin–diatomite PCM systems.

### 9.2. Possibilities for Their Integration in Sustainable Energy Systems and Building Energy Efficiency

The integration of paraffin–diatomite composites into sustainable energy systems constitutes a promising strategy for enhancing the energy efficiency of buildings. The distinctive properties of these composites render them suitable for diverse applications in thermal energy management, facilitating substantial advancements in building performance and sustainability metrics.

The integration of paraffin with diatomite as a carrier matrix offers superior thermal energy storage capabilities. The substantial heat capacity of paraffin enables efficient absorption and release of thermal energy, thereby mitigating temperature fluctuations within buildings and enhancing thermal comfort [218]. This characteristic is advantageous for passive heating and cooling strategies, allowing buildings to more effectively manage energy loads and sustain a consistent indoor temperature [229].

Diatomite, a naturally abundant material, significantly contributes to the sustainable development of these composites. Utilizing such materials reduces reliance on synthetic alternatives, thereby promoting environmentally friendly construction practices [102]. The integration of diatomite with paraffin can improve the mechanical stability of the composite, ensuring durability and longevity in construction applications [212].

The integration of paraffin–diatomite composites has the potential to substantially enhance the energy efficiency of heating, ventilation, and air conditioning (HVAC) systems. Empirical research indicates that the incorporation of these composites into construction materials can result in energy savings ranging from 8.3% to 25.1% during colder seasons, thereby presenting a viable strategy for reducing energy consumption in buildings [230]. Furthermore, the application of phase change materials (PCM) in conjunction with renewable energy sources, such as solar heating systems, optimizes energy utilization and maximizes efficiency [231].

Paraffin–diatomite composites play an essential role in load shifting by storing surplus thermal energy generated during the day and releasing it during periods of high demand or when renewable energy sources are less available. This characteristic is particularly significant in smart buildings, as it enhances their ability to interact efficiently with the power grid and reduce peak demand [104,232].

The thermal inertia afforded by paraffin–diatomite composites serves to diminish the operational demands placed on HVAC systems. By attenuating indoor temperature fluctuations, these composites enhance the efficiency of HVAC equipment and may facilitate the development of smaller, less energy-intensive systems [229]. This advancement contributes to reduced operating costs and a diminished carbon footprint for buildings.

While the successful integration of paraffin–diatomite composites into sustainable energy systems offers numerous advantages, several challenges must be addressed.

Paraffin–diatomite composites possess the capacity to absorb moisture, a factor that may influence their thermal properties and mechanical integrity. It is essential to regulate humidity levels within buildings to preserve the efficacy of these materials [45]. To address this concern, the implementation of advanced protective coatings or treatment processes may be required.

The processes involved in the production of high-quality composites are often intricate and costly, potentially limiting their widespread adoption. It is imperative to develop efficient manufacturing techniques that effectively balance cost, quality, and scalability, thereby ensuring the economic feasibility of these materials for mass production [102].

The thermal performance of paraffin–diatomite composites is subject to variation based on factors such as composition, production methods, and environmental conditions. Further research is required to establish standardized performance indicators and testing protocols to ensure reliability across diverse applications and conditions [218].

The integration of paraffin–diatomite composites into existing building systems may pose logistical challenges. It is imperative to undertake meticulous planning to evaluate building designs and energy systems, thereby facilitating the effective incorporation of these advanced materials [231].

The integration of paraffin–diatomite composites into sustainable energy systems represents a significant opportunity to enhance the energy efficiency of buildings. These composites possess unique properties that offer substantial advantages in thermal energy management, contributing to reduced energy consumption and improved indoor comfort, while also supporting environmental sustainability objectives. Nevertheless, for these composites to achieve widespread adoption in energy-efficient building projects, it is essential to address challenges related to moisture sensitivity, production complexity, and integration. Continued research and development will be instrumental in realizing the full potential of these innovative materials.

### 9.3. Directions for Further Research

The application of paraffin–diatomite composites in thermal energy storage presents significant potential; however, further investigation is required to optimize their properties and applications. Several areas necessitate detailed examination, including nanotechnology, the development of hybrid materials, and cost optimization.

The discipline of nanoengineering presents significant opportunities to enhance the thermal properties of paraffin–diatomite composites through the incorporation of nanomaterials. For instance, the integration of carbon nanotubes (CNT) or graphene into the composite matrix can markedly improve both thermal conductivity and mechanical strength. Empirical evidence indicates that the application of multi-walled carbon nanotubes (MWCNT) in paraffin-based systems results in a substantial augmentation of thermal energy storage capacity, attributable to their efficient heat transfer characteristics [165]. Continued investigation into various nanocomposite types may yield innovative solutions that further optimize the performance of paraffin and diatomite mixtures.

Research into the development of nanostructured diatomite composites, which function as effective fillers, holds significant promise. For instance, nano-Bi2MoO6/diatomite composites have been shown to enhance photocatalytic activity while also facilitating energy storage, thereby illustrating the versatility of this approach [233]. This field could further benefit from systematic investigations into the effects of varying nanoparticle sizes and distributions on thermal properties and stability.

Hybrid materials that integrate paraffin–diatomite composites with additional substances, such as bio-based polymers or alternative mineral fillers, have the potential to enhance thermal properties and sustainability. The incorporation of modified mineral fillers can substantially influence water absorption and mechanical properties, as evidenced by recent studies [44]. Investigating suitable hybrid combinations may facilitate the development of composites with improved properties, specifically tailored for applications in energy efficiency systems within buildings.

Research on microcapsules with hybrid shells composed of polymers and diatomite shows significant promise, as evidenced by the synthesis of micro-PCM with a shell comprising melamine, urea, and formaldehyde (MUF) in conjunction with diatomite. These microcapsules are utilized for thermal energy storage and demonstrate enhanced mechanical properties, rendering them suitable for practical applications in temperature regulation [105]. Investigating various core and shell configurations for microencapsulation could further improve the stability and performance of paraffin-based storage solutions.

Profitability continues to pose a significant challenge to the extensive adoption of paraffin–diatomite composites. Further investigation is required into material sourcing, processing methodologies, and formulations to decrease production costs. For instance, the production process, encompassing calcination and the determination of optimal mixing ratios, should be assessed to reduce energy consumption during production [43].

Research into the utilization of industrial waste, such as slag, in the development of composites may offer a cost-effective alternative and promote recycling while enhancing the properties of the composites [216]. Investigations into the scalability of production methods, including in situ polymerization or solvent-free mixing techniques, may also reveal strategies to reduce costs and increase efficiency [234].

From an economic standpoint, it is imperative to systematically integrate life cycle assessments to evaluate the trade-offs between cost and performance associated with the use of paraffin–diatomite composites in energy-efficient buildings. Such assessments can yield valuable insights, thereby rendering these materials a more cost-effective option in energy management solutions.

Significant opportunities exist for the advancement of paraffin–diatomite composite materials through the application of nanotechnology, the development of hybrid materials, and the implementation of cost optimization strategies. Targeted research in these domains could substantially enhance their performance in thermal energy storage systems, thereby increasing their utility in sustainable energy practices within the construction sector. Furthermore, interdisciplinary collaboration among materials science, chemistry, and engineering will be essential to fully realize the potential of these innovative composite materials.

The incorporation of diatomite with paraffin-based phase change materials (PCMs) presents considerable potential for the advancement of thermal energy storage technologies. Nonetheless, to fully harness this potential, it is imperative to address several research priorities and technical challenges.

Future research should prioritize enhancing the sorption capacity of diatomite with paraffins. Although previous studies have highlighted the potential of diatomite as a carrier for phase change materials (PCMs), the sorption efficiency remains inadequate. Prior investigations have reported absorption ratios ranging from approximately 35% to 50% for paraffin in diatomaceous composites [88]. This underscores the need to optimize the pore structure and chemical modifications of diatomite to improve its absorption capabilities. Furthermore, investigating alternative paraffins and blends could result in materials with superior thermal stability and latent heat values, thereby enhancing thermal performance [235,236].

Another critical research priority involves addressing the stability of diatomite/paraffin composites during phase transitions. Studies have demonstrated that phase separation and leakage of PCMs during thermal cycling remain significant challenges, with numerous existing materials susceptible to leakage when exposed to heat [85,237]. Progress in encapsulation techniques, such as emulsion polymerization or novel impregnation methods, is essential to ensure the long-term stability and efficacy of PCMs [238]. Additionally, exploring new methods for the structural reinforcement of the composites may also contribute to mitigating these issues.

Future research should prioritize the investigation of diatomite/paraffin composites in practical applications, such as construction materials and cooling technologies. The potential of these materials for thermal insulation in buildings or as heat sinks for electronic devices presents a promising area for further exploration. The inherent physical and chemical properties of diatomite indicate its significant potential as a low-density thermal energy storage medium, especially when combined with paraffins, which exhibit advantageous thermal properties [45,101,151]. Additionally, research should examine the scalability and economic feasibility of these composites for widespread implementation in construction and cooling applications.

To overcome the technical challenges associated with synthesizing effective diatomite/paraffin composites, refining the preparation and impregnation processes is essential. Techniques such as vacuum impregnation and fusion adsorption have demonstrated potential [236,238]. By optimizing these methods, it is feasible to enhance the uniform distribution of paraffin within the diatomite structure, thereby improving thermal performance and minimizing leakage.

Research indicates that the integration of diatomite with other materials, such as expanded graphite or organic matrices, can result in hybrid composites with enhanced thermal performance [239,240]. The addition of reinforcing agents may improve not only thermal conductivity but also mechanical integrity, which is essential for practical applications. Investigating combinations with other minerals or polymers can yield versatile materials tailored to specific thermal storage requirements.

Conducting comprehensive experimental evaluations of engineered diatomite/PCM composites under diverse thermal conditions and mechanical stresses is imperative. The development of standardized testing protocols to assess performance metrics, such as thermal cycling resilience, latent heat storage capacity, and structural integrity, will yield critical insights into the practical applicability of these materials [187,241]. Ensuring optimal performance of these materials in real-world conditions is essential for fostering industry confidence and promoting broader adoption.

Advancements in the field of diatomite/paraffin composites can be significantly fostered by prioritizing the enhancement of thermal properties, ensuring stability, and exploring diverse applications. Concurrently, overcoming technical challenges through optimized preparation and validation is essential.

Figure 1 and Figure 5 were generated using artificial intelligence (AI) tools to visually illustrate the conceptual framework and material correlations discussed in this study. The images were created exclusively for scientific visualization purposes and do not represent real experimental data.

## Figures and Tables

**Table 1:** Physicochemical properties of popular paraffins [56,57,58,59].

Substance	Melting Point [°C]	Heat of Fusion [kJ/kg]	Thermal Conductivity [W/m K]
n-Hexadecane (C16)	≈17–19	≈200–220	≈0.20–0.30
n-Octadecane (C18)	≈26–29	244.8	≈0.20–0.34
n-Eicosane (C20)	≈36–38	240–245	≈0.20–0.35
n-Docosane (C22)	≈40–42	≈242–248	(solid) ≈ 0.12–0.18; (liquid) ≈ 0.21–0.24
Commercial paraffin wax (RT-series)	various (e.g., RT21 ≈ 21 °C, RT27 ≈ 27 °C, RT35 ≈ 35 °C)	usually 130–210 (depending on composition and purity)	usually 0.20–0.40

**Table 2:** Characteristics of diatomite as a PCM carrier—comparison with other carrier materials [91,92,93,94,95,96].

**Material (carrier)**	Diatomite/diatomaceous earth	Zeolites (e.g., Na-clinoptilolite, synthetic)	Expanded perlite	Silica (porous silica, mesoporous SiO_2_, fumed silica)	Clays/bentonite/ vermiculite
**Structure/key features**	very porous, high specific surface area, low density; natural, easily modifiable	cardboard-like crystal lattice, micro-/mesopores; selective adsorption interactions	porous silicate material in granule form (low density)	controlled pores (meso-), high specific surface area, possibility of surface functionalization	layered minerals, varying porosity; bentonite—high swelling capacity and ion exchange ability
**Typical PCM content [wt.%]**	~40–70	~20–60 (depending on pores and modifications)	~40–60 (typically ~50–55 without leakage with coatings)	~10–50 (mesoporous carriers usually have a lower mass fraction of PCM than large porous minerals)	~20–60 (a lot depends on the type of clay)
**Thermal conductivity**	low to moderate, depending on porosity (~0.07–0.2 W/m·K)	usually low to moderate; can be hybridized with graphite/metals	low (~0.1 W/m·K)—graphite/EG/metal is often added	usually low, but the addition of nanostructures and conductors (CNT, graphite) increases conductivity	low to moderate; often worse than silica/zeolites—possible improvements through modification
**Leak resistance**	good if the PCM is inside the pores; required coating modifications for dynamic cycles	good; the crystalline structure limits liquid migration, but pore and impregnation control is required	good after coating/modification; without modification, there is a risk of leakage during long cycles	very good (strong capillarity and adsorption), especially with sol–gel, MCM-41, SBA-15	good for physical adsorption; no chemical reactions with fatty acids were observed in the studies
**Chemical compatibility/stability**	chemically inert to paraffins, after calcination changes its sorption and thermal stability	very good compatibility, thermally/chemically stable; sometimes surface modification is required (compatibility with acids/bases)	chemically stable, easy to integrate into building materials	good chemical stability, the surface can be functionalized for better compatibility with PCM (e.g., PEG)	some types require drying/calcination
**Typical applications/notes**	construction (fillings, panels), lightweight thermo-accumulative granules; low price, natural	specialized applications (cascade TES systems, adsorption-PCM hybrids)	in construction (composites, panels, lightweight concrete), low cost	applications requiring precise control of morphology and cycles; often in the form of nanocomposites/ssPCM	construction applications and composites; easy to mix with cement/gypsum

**Table 3:** Comparison of physical, chemical, and hybrid preparation techniques for paraffin–diatomite composites, including process parameters and thermal performance indicators [42,91,103,112,113,114,115,116,117,118].

Typical Process Conditions	PCM Loading [%]	Thermal Conductivity Improvement [%]	Latent Heat Retention [%]	Leakage Resistance	Advantages	Limitations
60–80 °C, reduced pressure or capillary forces; impregnation 2–6 h	50–70	10–25	85–95	good	simple process, no chemical reagents, good shape stability	limited interfacial compatibility, possible incomplete pore filling
Calcination 450–650 °C; silanization with silane coupling agents	55–75	25–40	90–97	very good	enhanced interfacial adhesion, reduced moisture absorption, higher stability	requires controlled processing, potential cost increase
Vacuum impregnation + additive dispersion (graphene 1–3 wt.%)	45–65	40–70	88–95	excellent	strong increase in thermal conductivity, mechanical reinforcement	complex preparation, higher cost, possible aggregation of fillers

**Table 4:** Summary of current contradictions and potential research directions in paraffin–diatomite composite PCMs [45,112,115,120,122,161,184,187,219,220,221,222,223,224,225,226,227,228].

Research Contradiction	Observed Issue	Recommended Direction
thermal conductivity vs. latent heat	agglomeration of fillers reduces the PCM content	use surface-functionalized nanofillers
surface modification vs. cycling durability	degradation of silane groups over cycles	develop thermally stable coupling agents
porosity vs. mechanical strength	high porosity weakens the structure	optimize pore-size distribution
cost vs. scalability	complex chemical modification increases cost	focus on low-cost hybridization routes

## Data Availability

No new data were created or analyzed in this study. Data sharing is not applicable to this article.
